# VE-Cadherin–Mediated Epigenetic Regulation of Endothelial Gene Expression

**DOI:** 10.1161/CIRCRESAHA.117.312392

**Published:** 2017-12-12

**Authors:** Marco F. Morini, Costanza Giampietro, Monica Corada, Federica Pisati, Elisa Lavarone, Sara I. Cunha, Lei L. Conze, Nicola O’Reilly, Dhira Joshi, Svend Kjaer, Roger George, Emma Nye, Anqi Ma, Jian Jin, Richard Mitter, Michela Lupia, Ugo Cavallaro, Diego Pasini, Dinis P. Calado, Elisabetta Dejana, Andrea Taddei

**Affiliations:** From the IFOM, FIRC Institute of Molecular Oncology, Milan, Italy (M.F.M., C.G., M.C., F.P., E.D., A.T.); Department of Biomedicine, University of Basel, Switzerland (M.F.M.); Laboratory of Thermodynamics in Emerging Technologies, Department of Mechanical and Process Engineering, ETH Zurich, Switzerland (C.G.); Cogentech, Milan, Italy (F.P.); Department of Experimental Oncology (E.L., D.P.) and Unit of Gynecological Oncology Research (M.L., U.C.), European Institute of Oncology, Milan, Italy; Department of Immunology, Genetics and Pathology, Rudbeck Laboratory, Uppsala University, Sweden (S.I.C., L.L.C., E.D.); Peptide Chemistry (N.O., D.J.), Structural Biology (S.K., R.G.), Experimental Histopathology (E.N.), Bioinformatics & Biostatistics Department (R.M.), and Immunity and Cancer Laboratory (D.P.C., A.T.), The Francis Crick Institute, London, United Kingdom; Center for Chemical Biology and Drug Discovery, Departments of Pharmacological Sciences and Oncological Sciences, Tisch Cancer Institute, Icahn School of Medicine at Mount Sinai, New York, NY (A.M., J.J.); and Department of Oncology and Hemato-Oncology, University of Milan, Italy (E.D.).

**Keywords:** blood vessels, cadherin, cell differentiation, endothelial cells, polycomb-group proteins

## Abstract

**Rationale:**

The mechanistic foundation of vascular maturation is still largely unknown. Several human pathologies are characterized by deregulated angiogenesis and unstable blood vessels. Solid tumors, for instance, get their nourishment from newly formed structurally abnormal vessels which present wide and irregular interendothelial junctions. Expression and clustering of the main endothelial-specific adherens junction protein, VEC (vascular endothelial cadherin), upregulate genes with key roles in endothelial differentiation and stability.

**Objective:**

We aim at understanding the molecular mechanisms through which VEC triggers the expression of a set of genes involved in endothelial differentiation and vascular stabilization.

**Methods and Results:**

We compared a VEC-null cell line with the same line reconstituted with *VEC* wild-type cDNA. VEC expression and clustering upregulated endothelial-specific genes with key roles in vascular stabilization including *claudin-5*, vascular endothelial-protein tyrosine phosphatase (*VE-PTP*), and von Willebrand factor (*vWf*). Mechanistically, VEC exerts this effect by inhibiting polycomb protein activity on the specific gene promoters. This is achieved by preventing nuclear translocation of FoxO1 (Forkhead box protein O1) and β-catenin, which contribute to PRC2 (polycomb repressive complex-2) binding to promoter regions of *claudin-5*, *VE-PTP*, and *vWf*. VEC/β-catenin complex also sequesters a core subunit of PRC2 (Ezh2 [enhancer of zeste homolog 2]) at the cell membrane, preventing its nuclear translocation. Inhibition of Ezh2/VEC association increases Ezh2 recruitment to *claudin-5*, *VE-PTP*, and *vWf* promoters, causing gene downregulation. RNA sequencing comparison of VEC-null and VEC-positive cells suggested a more general role of VEC in activating endothelial genes and triggering a vascular stability-related gene expression program. In pathological angiogenesis of human ovarian carcinomas, reduced VEC expression paralleled decreased levels of claudin-5 and VE-PTP.

**Conclusions:**

These data extend the knowledge of polycomb-mediated regulation of gene expression to endothelial cell differentiation and vessel maturation. The identified mechanism opens novel therapeutic opportunities to modulate endothelial gene expression and induce vascular normalization through pharmacological inhibition of the polycomb-mediated repression system.

## Abstract 2

Supplemental Digital Content is available in the text.

Several human pathological conditions are characterized by deregulated angiogenesis leading to the formation of unstable blood vessels.^1^ Abnormal angiogenesis is also a hallmark of cancer. Solid tumors get their nourishment from newly formed vessels, which, however, present several structural abnormalities such as wide and irregular interendothelial junctions.^2^

VEC (vascular endothelial cadherin), the main component of endothelial adherens junctions (AJs), plays a major role in the process of vessel maturation and stability. This molecule, specifically expressed by endothelial cells (ECs), mediates homophilic adhesion at cell–cell contacts regulating vascular permeability.^3^ VEC is indispensable for vascular maturation and inhibition of vascular regression.^3^ VEC clustering at AJs triggers intracellular signals inducing contact inhibition of cell growth, protection from apoptosis, cell polarity, and inhibition of migration.^4^

In previous work, we found that VEC expression and clustering at cell–cell contacts relieve the inhibitory effect of the FoxO1 (Forkhead box protein O1)/β-catenin complex on the expression of claudin-5, an endothelial-specific TJ (tight junction) protein,^5^ acting via the removal of a transcriptional repression mechanism.

The reversible nature of *claudin-5* gene repression suggested the involvement of PcG (polycomb group) proteins and epigenetic mechanisms in VEC-mediated regulation of *claudin-5* expression. Indeed, PcG proteins control the induction of reversible states of epigenetic silencing in most multicellular organisms, including humans, and regulate several developmental decisions by silencing genes involved in stem cell differentiation and specification of cellular identities.^6,7^ Their activity is exerted through 2 multiprotein complexes called PRC (polycomb repressive complex)1 and PRC2.^6^ PRC2 core subunits are Ezh (enhancer of zeste homolog)2, Suz (suppressor of zeste)12, Eed (embryonic ectoderm development), and Rbap (retinoblastoma-binding protein)48. Ezh2 is the catalytic subunit containing a SET [Su(var)3-9, enhancer of zeste and trithorax] domain, which harbors the active site for histone H3 trimethylation on lysine 27, although Suz12 and Eed association is needed for optimal enzymatic activity.^8^ According to the canonical model, H3K27me3 (histone H3 trimethylated on lysine 27) is a silencing histone mark promoting the recruitment of PRC1 selectively at genes that are targeted for repression. PRC1 includes Ring1B, which mediates monoubiquitylation of histone H2A on lysine 119^9^ blocking gene expression by multiple mechanisms such as chromatin compaction, inhibition of transcriptional initiation or elongation, recruitment of transcriptional inhibitors, and block of the binding of key activators.^6,10^

A fundamental step in PRC-mediated transcriptional repression is the ability of such complexes to be recruited specifically at target genes. In Drosophila, this is accomplished by polycomb response elements, clusters of DNA-binding sites for proteins that associate with PRC2 and PRC1.^11^ Mammalian polycomb response elements have not been identified yet, and many aspects of polycomb recruitment still remain obscure.

Here, we report that VEC-mediated inhibition of FoxO1/β-catenin nuclear accumulation triggers a transcriptional program of endothelial differentiation and maturation. PcG proteins exert an essential role in such program by targeting the promoters of key endothelial stability genes *claudin-5*, vascular endothelial-protein tyrosine phosphatase (*VE-PTP*), and von Willebrand factor (*vWf*), causing their repression. VEC can inhibit such mechanism in multiple ways, by preventing polycomb binding to gene promoters through restriction of the nuclear localization of the FoxO1/β-catenin complex and by sequestering a fraction of Ezh2 at the plasma membrane.

These observations are supported by in vivo data in newborn mice treated with Ezh2 inhibitor UNC1999. Furthermore, dismantling of VEC clusters during pathological angiogenesis in human ovarian carcinomas is accompanied by the downregulation of Claudin-5 and VE-PTP.

These data highlight a key role of PcG proteins in the regulation of EC gene expression and open novel therapeutic opportunities to induce vascular normalization through pharmacological inhibition of the polycomb-mediated repression system.

## Methods

Detailed Methods section is available in the Online Data Supplement.

The data that support the findings of this study are available from the corresponding author on reasonable request.

## Results

### VEC Clustering Triggers an Endothelial-Specific Transcription Program

**Figure 1.**
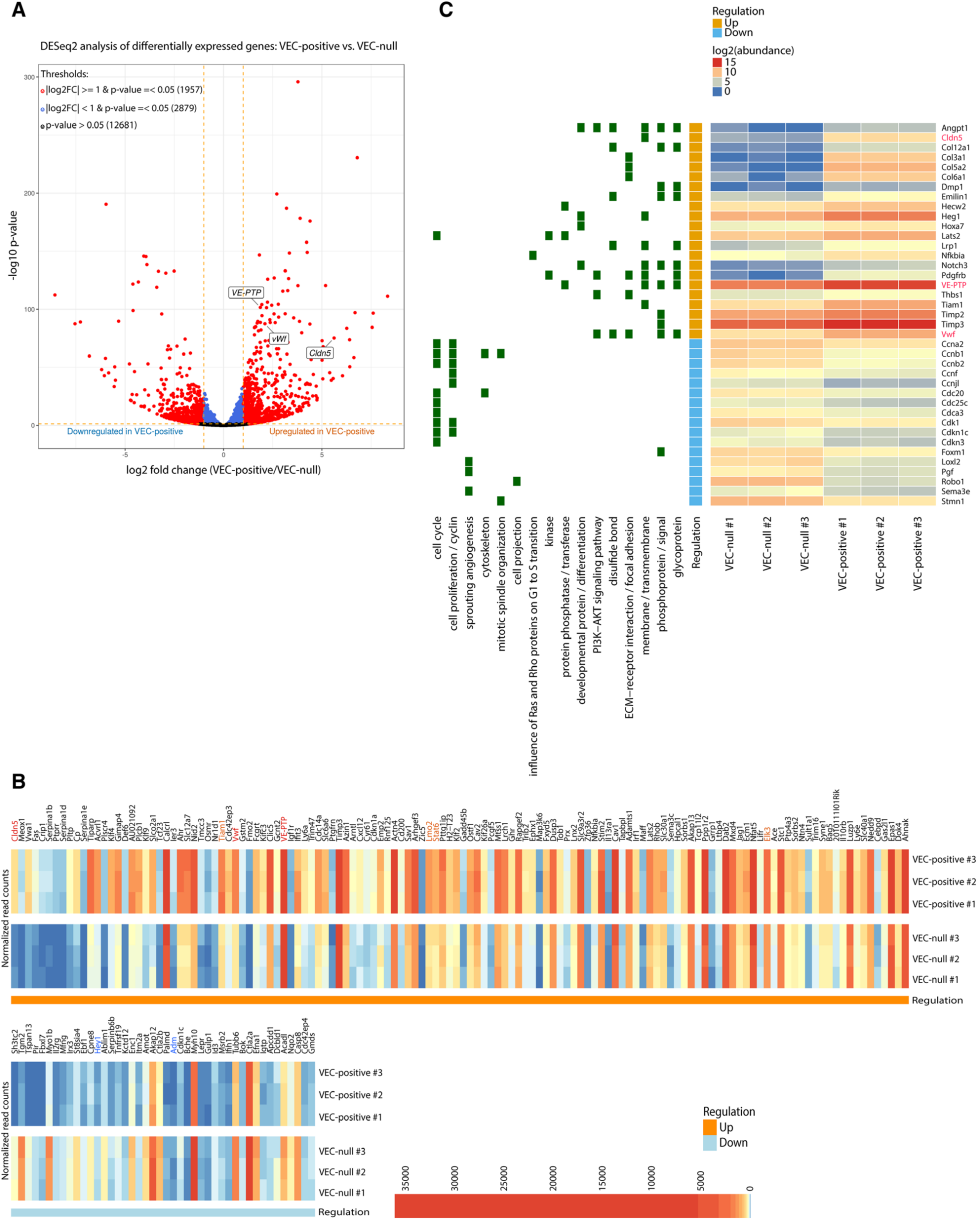
**Transcriptome profile determined by VEC (vascular endothelial cadherin) expression and clustering.** **A**, Volcano plot showing the magnitude of differential expression between VEC-positive and VEC-null endothelial cells (ECs). Each dot represents 1 gene with detectable expression in both cell types. The horizontal dashed line (orange) together with the vertical lines (orange) mark thresholds used (*P* value ≤0.05 and |log2FC| ≥1) to define a gene as differentially regulated in VEC-positive (red). Genes that only passed threshold *P* value ≤0.05 are depicted in blue. Dots representing *claudin*-5, vascular endothelial-protein tyrosine phosphatase (*VE-PTP*), and von Willebrand factor (*vWF*) are labeled in the figure. **B**, Heat-map showing the expression pattern of significantly differentially expressed endothelial genes (*P* value ≤0.05 and |log2FC| ≥1) within and between biological replicates. Endothelial genes upregulated (red/orange) or downregulated (blue) in VEC-positive cells which were further investigated in this study are highlighted in the figure. Genes are displayed in decreasing |log2FC| order (left to right). **C**, Heat-map showing normalized abundance of significantly changing genes across all samples. Genes belonging to selected functionally enriched terms are highlighted in green on the left of the plot. *Claudin-5*, *VE-PTP*, and *vWf* genes are highlighted in red. In (**B**) and (**C**), VEC-positive numbers 1/2/3 represent biological replicates in VEC-positive cells, whereas VEC-null numbers 1/2/3 represent biological replicates in VEC-null cells.

To investigate whether VEC was able to upregulate other endothelial-specific genes besides *claudin-5*,^5^ we performed an RNA sequencing comparative analysis of a mouse *VEC*-null cell line (VEC-null) and the same line reconstituted with *VEC* wild-type cDNA (VEC-positive). Several genes were upregulated by VEC expression and clustering (Figure 1A). Taking advantage of previously published data on endothelial gene expression,^12^ we selected a list of endothelial genes whose expression was induced or repressed by VEC (Figure 1B; Online Tables I and II). Further validation of RNA sequencing data was performed by quantitative real-time polymerase chain reaction of *VE-PTP*,^13^ *vWf*,^14^ T-cell lymphoma invasion and metastasis-1 (*Tiam1*),^15^ LIM domain only-2 (*Lmo2*),^16^ signal transducer and activator of transcription-6 (*Stat6*)^17^ and *Elk3* (ETS domain containing protein)^18,19^ among VEC-induced genes, hes-related family bHLH transcription factor with YRPW motif-1 (*Hey1*)^20^ and adrenomedullin (*Adm*)^21^ among VEC-repressed genes, and platelet/endothelial cell adhesion molecule-1 (*Pecam1*)^22^ and SRY (sex determining region Y)-box-18 (*Sox18*)^19^ as genes not influenced by VEC expression (Online Figures IA and IIA through IIC). Similar transcriptional changes were induced by VEC clustering when comparing human dermal microvascular ECs (HMEC-1) in sparse and confluent conditions (Online Figure IID through IIG). Interestingly, VEC also seemed to coordinate the expression of several genes promoting vascular stability, according DAVID Functional Annotation Clustering tool.^23^ For instance, we observed a general downregulation of genes involved in cell proliferation and sprouting angiogenesis, while extracellular matrix interaction and cell–cell adhesion were promoted (Figure 1C).

Within VEC-upregulated endothelial genes, we selected the tyrosine phosphatase *VE-PTP*^13^ and the extracellular matrix protein *vWf*^14^ for further studies. These 2 genes were selected by virtue of their endothelial specificity and their described role in the induction of vascular stability.^13,14^ As reported for *claudin-5*,^5^ both genes were strongly upregulated by VEC expression and clustering (Online Figure IA). A mutant version of VEC composed of the cadherin cytoplasmic tail fused to the transmembrane and extracellular domains of IL-2 (interleukin-2) receptor α-chain (IL2-VEC),^5^ which is unable to cluster at cell–cell contacts, did not upregulate these genes, confirming the need of VEC clustering for this effect (Online Figure III). As for *claudin-5*,^5^ FoxO1 and β-catenin activity inhibited *VE-PTP* and *vWf* expression. Infection of confluent VEC-positive cells with an adenovirus encoding a constitutively active form of FoxO1 (FKHR-TM [Forkhead transcription factor triple mutant])^24^ significantly downregulated both *VE-PTP* and *vWf* expression (Online Figure IB). Similarly, increasing endogenous FoxO1 activity by LY294002-mediated inhibition of PI3K (phosphatidylinositol 3 kinase) reduced FoxO1-Ser256 (serine 256) phosphorylation level (Online Figure IC, upper) and downregulated both genes under study (Online Figure IC). β-Catenin associates with FoxO1 and stabilizes its binding to *claudin-5* promoter.^5^ The expression of a stabilized version of β-catenin (ΔN-β-catenin)^5^ led to a marked downregulation of both *VE-PTP* and *vWf* genes (Online Figure ID), suggesting a regulatory mechanism similar to that of *claudin-5*. A promoter analysis spanning from 6000 bp upstream to 500 bp downstream of the transcription start site (TSS) of *VE-PTP* and *vWf* genes identified a series of paired Tcf (T-cell factor)/β-catenin/FoxO1-binding sites localized in 3 different regions on both promoters (Online Figure IE). Quantitative chromatin immunoprecipitation showed that FoxO1 binds all 3 regions in both promoters (Online Figure IF). Binding occurred only in confluent VEC-null and not in confluent VEC-positive cells, correlating with gene repression. Furthermore, β-catenin also bound all identified regions with different affinity (Online Figure IG), consistently with what previously reported.^5^ Thus, VEC upregulates the endothelial-specific genes *vWf* and *VE-PTP* through a mechanism similar to *claudin-5* upregulation.

### *Claudin-5*, *VE-PTP*, and *vWf* Are Polycomb Target Genes

**Figure 2.**
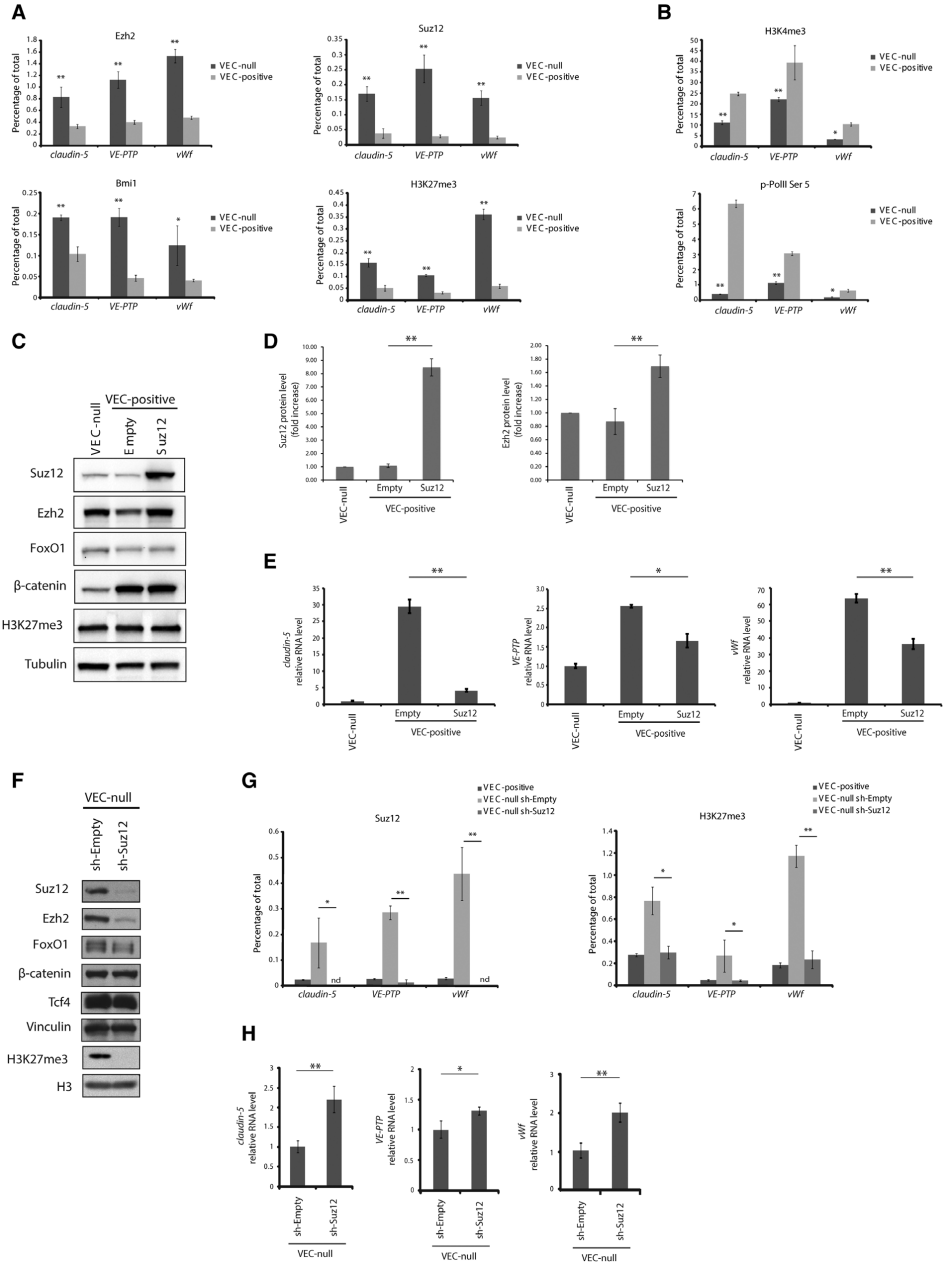
***Claudin-5*, vascular endothelial-protein tyrosine phosphatase (*VE-PTP*), and von Willebrand factor (*vWf*) are polycomb targets.** **A**, Quantitative real-time polymerase chain reaction (qRT-PCR) for the transcription start site (TSS) of *claudin-5*, *VE-PTP*, and *vWf* performed on endogenous Ezh (enhancer of zeste homolog)2-, Suz (suppressor of zeste)12-, Bmi1 (B lymphoma Mo-MLV insertion region 1)-, and H3K27me3 (histone H3 trimethylated on lysine 27)-bound chromatin immunoprecipitated from confluent VEC (vascular endothelial cadherin)-null and VEC-positive endothelial cells (ECs). **B**, qRT-PCR for the TSS of *claudin-5*, *VE-PTP*, and *vWf* performed on endogenous H3K4me3-bound and RNA polymerase II (p-PolII) Ser5–bound chromatin immunoprecipitated from confluent VEC-null and VEC-positive ECs. **C**, Western blot (WB) analysis of indicated proteins in extracts of confluent VEC-null and VEC-positive ECs upon Suz12 overexpression. **D**, Quantification of WB in (**C**). Suz12 and Ezh2 levels were normalized to tubulin. Columns are means±SEM of 3 independent experiments. **E**, qRT-PCR analysis of *claudin-5*, *VE-PTP*, and *vWf* expression in confluent VEC-null and VEC-positive ECs upon Suz12 overexpression. **F**, WB analysis of indicated proteins in extracts of confluent VEC-null ECs upon Suz12 knockdown (sh-Suz12). **G**, qRT-PCR for the TSS of *claudin-5*, *VE-PTP*, and *vWf* performed on endogenous Suz12- and H3K27me3-bound chromatin immunoprecipitated from confluent VEC-positive, VEC-null-sh-Empty, and VEC-null-sh-Suz12 ECs. **H**, qRT-PCR analysis of *claudin-5*, *VE-PTP*, and *vWf* expression in confluent VEC-null-sh-Empty and VEC-null-sh-Suz12 ECs. **A**, **B**, **G**, Levels of DNA are normalized to input, columns are means±SD of triplicates from a representative experiment. **C**, **F**, Tubulin and vinculin are the loading controls. **E**, **H**, Levels of mRNA are normalized to 18S; columns are means±SEM of triplicates from a representative experiment. In (**A**) and (**B**), *P<0.05; **P<0.01, *t* test VEC-null vs VEC-positive. In (**G**), *P<0.05; **P<0.01, *t* test VEC-null Sh-Empty vs VEC-null Sh-Suz12. In (**D**), (**E**), and (**H**), *P<0.05; **P<0.01, *t* test. nd indicates not detectable.

We then aimed at clarifying how the FoxO1/β-catenin complex might act on *claudin-5*, *VE-PTP*, and *vWf* genes to induce their repression. Given the endothelial specificity of the identified genes, we hypothesized the possible involvement of PcG proteins, a group of transcriptional regulators involved in cell specification, which mediate reversible inhibition of transcription.^6^ We performed quantitative chromatin immunoprecipitation for PcG proteins on the TSS of *claudin-5*, *VE-PTP*, and *vWf* genes. Gene TSSs displayed higher enrichment in the components of both PRC2 (Ezh2 and Suz12) and PRC1 (Bmi1; B lymphoma Mo-MLV insertion region 1) in confluent VEC-null compared with VEC-positive cells (Figure 2A, top and lower left). The mark of Ezh2 enzymatic activity, H3K27me3, also showed a similar enrichment pattern (Figure 2A, lower right), and quantitative chromatin immunoprecipitation for total histone H3 showed comparable enrichments at the analyzed regions in VEC-null and VEC-positive cells (Online Figure IV), ruling out the possibility that the higher H3K27me3 signal in VEC-null cells could be because of widespread higher histone density. PcG-mediated repression is counteracted by the activity of TrxG (trithorax group) proteins.^6^ As expected, *claudin-5*, *VE-PTP*, and *vWf* TSSs showed a higher enrichment in H3K4me3, marker of TrxG activity and gene activation, in confluent VEC-positive cells than in VEC-null cells (Figure 2B, upper). RNA polymerase II phosphorylated on Ser5, a modification needed for the enzyme to escape the promoter and transcribe the gene, was also increased at the TSSs of genes in confluent VEC-positive cells (Figure 2B, lower).

When we overexpressed the PRC2 member Suz12 in confluent VEC-positive cells using lentiviral-mediated gene delivery (Figure 2C), *claudin-5*, *VE-PTP*, and *vWf* expression was reduced (Figure 2E). PRC2 proteins are known to promote each other’s stability by physical interaction.^8^ Suz12 overexpression led to an almost 2-fold increase in Ezh2 protein level (Figure 2D). The promoter region of *claudin*-5 (3169 bp upstream of the coding sequence; Online Figure VA), comprising all 3 regions of paired Tcf/β-catenin/FoxO1-binding sites^5^ was cloned upstream of the firefly luciferase gene. As expected, transfection of this reporter in confluent VEC-null and VEC-positive cells showed higher luciferase activity in the presence of VEC expression and clustering (Online Figure VB). Suz12 overexpression in VEC-positive ECs significantly decreased luciferase expression (Online Figure VC). *Lmo2* and *Stat6* were also downregulated by Suz12 overexpression (Online Figure VIA), suggesting that the identified mechanism might regulate a wider set of endothelial genes. *Hey1* expression was induced by Suz12 (Online Figure VIB), likely as a result of polycomb-mediated downregulation of a *Hey1* gene repressor, while *Pecam1* and *Sox18* levels were not altered (Online Figure VIC). Conversely, Suz12 knockdown led to a marked decrease in Ezh2 and to the abrogation of detectable H3K27me3 levels (Figure 2F). FoxO1 protein levels were ≈30% lower on Suz12 knockdown (Figure 2F), although FoxO1 mRNA expression was unchanged (Online Figure VII). Suz12 knockdown abolished Suz12 and H3K27me3 signal at the TSSs of *claudin-5*, *VE-PTP*, and *vWf* genes (Figure 2G) causing a partial reactivation of their expression (Figure 2H). The incomplete rescue of expression might be because of the lack of specific gene activators missing in a VEC-null context or to PRC1 activity keeping genes partially repressed in the absence of PRC2.^25^ Furthermore, Suz12 knockdown induced an increase in *claudin-5* promoter activity as assessed by luciferase reporter assay (Online Figure VD).

### FoxO1/β-Catenin Enhance PcG Protein Binding to *Claudin-5, VE-PTP*, and *vWf* Promoters

**Figure 3.**
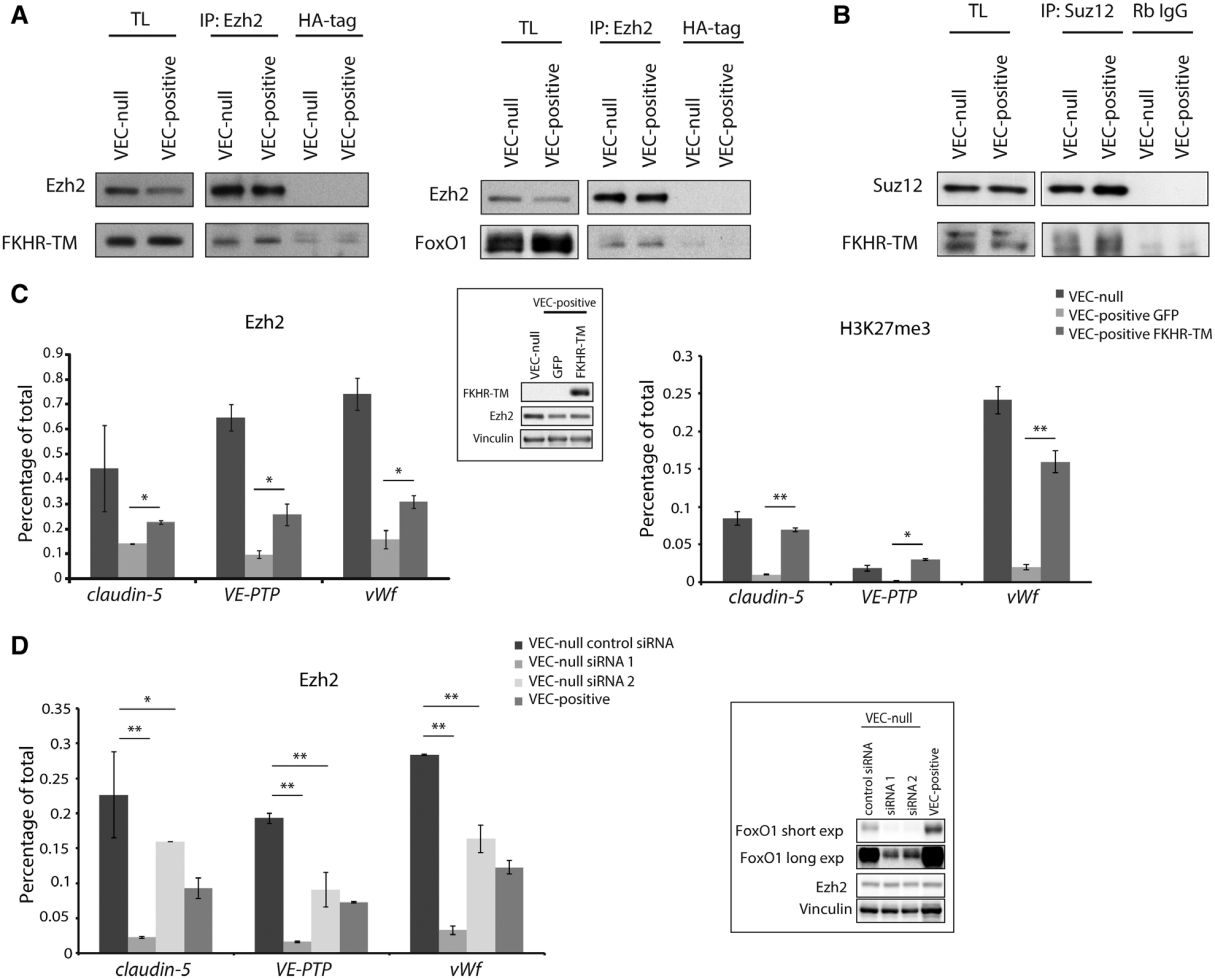
**FoxO1 (Forkhead box protein O1) enhances PcG (polycomb group) protein association to *claudin-5*, vascular endothelial-protein tyrosine phosphatase (*VE-PTP*), and von Willebrand factor (*vWf*) promoters.** **A**, **B**, Coimmunoprecipitation and Western blot (WB) of endogenous Ezh (enhancer of zeste homolog)2 or Suz (suppressor of zeste)12 and endogenous FoxO1 or FKHR-TM (Forkhead transcription factor triple mutant) from extracts of confluent VEC (vascular endothelial cadherin)-null and VEC-positive endothelial cells (ECs) or the same cells types expressing FKHR-TM (myc-tagged). **C**, Quantitative real-time polymerase chain reaction (qRT-PCR) for the transcription start site (TSS) of *claudin-5*, *VE-PTP*, and *vWf* performed on endogenous Ezh2- and H3K27me3 (histone H3 trimethylated on lysine 27)-bound chromatin immunoprecipitated from confluent VEC-null and VEC-positive ECs expressing either FKHR-TM or GFP (green fluorescent protein; negative control). Inset: WB analysis of FKHR-TM and Ezh2. **D**, qRT-PCR for the TSS of *claudin-5*, *VE-PTP*, and *vWf* performed on endogenous Ezh2-bound chromatin immunoprecipitated from confluent VEC-positive or VEC-null ECs transfected with control siRNA or with 2 siRNAs targeting FoxO1 mRNA. Inset: WB analysis of FoxO1 and Ezh2. Two different film exposure timings are shown for FoxO1. **C**, **D**, Vinculin is the loading control. Levels of DNA are normalized to input, columns are means±SD of triplicates from a representative experiment. In (**C**), *P<0.05; **P<0.01, *t* test VEC-positive GFP vs VEC-positive FKHR-TM. In (**D**), *P<0.05; **P<0.01, *t* test VEC-null control siRNA vs VEC-null siRNA1 or VEC-null siRNA2. IP indicates immunoprecipitation; and TL, total cell lysate.

We then hypothesized that the FoxO1/β-catenin complex could positively modulate polycomb interaction with the promoters of the identified genes. By coimmunoprecipitation, we found that constitutively active FKHR-TM or endogenous FoxO1 interact with Ezh2 (Figure 3A). Another member of PRC2, Suz12, also coimmunoprecipitated with FKHR-TM (Figure 3B). FKHR-TM overexpression in VEC-positive confluent cells increased PcG protein binding to the TSS of *claudin-5*, *VE-PTP*, and *vWf* genes, as shown by Ezh2 quantitative chromatin immunoprecipitation (Figure 3C, left). Consequently, H3K27me3 repressive histone mark was increased (Figure 3C, right). This is consistent with gene downregulation observed on FKHR-TM overexpression (Online Figure IB). No increase in Ezh2 protein levels was induced by FKHR-TM (Figure 3C inset), proving that the augmented Ezh2 enrichment was independent of protein upregulation. Conversely, FoxO1 knockdown strongly reduced Ezh2 recruitment at target sites in VEC-null cells to levels comparable to those detected in VEC-positive cells, or even lower (Figure 3D), in the absence of any Ezh2 downregulation (Figure 3D inset). Interestingly, this effect was dose dependent. SiRNA 2 was less efficient than siRNA 1 in knocking down FoxO1 (see FoxO1 long exp, Figure 3D inset), and this resulted in a weaker reduction of Ezh2 binding to gene TSSs.

Coexpression of Suz12 and FKHR-TM in VEC-positive confluent cells induced a stronger repression of *claudin-5* than the expression of FKHR-TM alone (75.3% versus 53.1%; Online Figure VIIIA and VIIIB), further supporting the hypothesis that FoxO1 acts in concert with polycomb activity.

**Figure 4.**
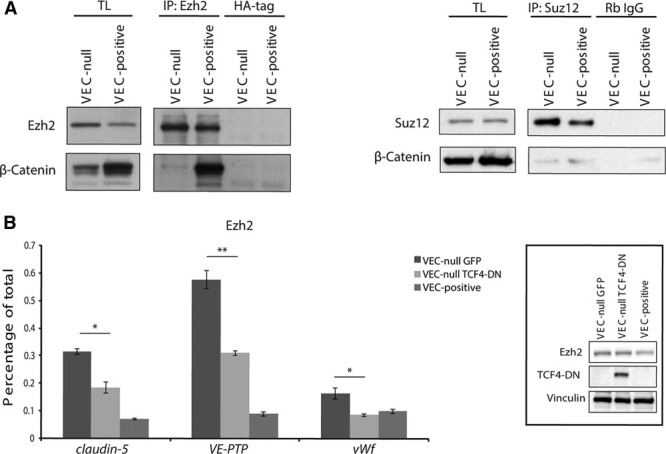
**β-Catenin stabilizes polycomb/DNA interaction on *claudin-5*, vascular endothelial-protein tyrosine phosphatase (*VE-PTP*), and von Willebrand factor (*vWf*) promoters.** **A**, Coimmunoprecipitation and Western blot (WB) analysis of endogenous Ezh (enhancer of zeste homolog)2 or Suz (suppressor of zeste)12 and β-catenin from extracts of confluent VEC (vascular endothelial cadherin)-null and VEC-positive endothelial cells (ECs). **B**, Quantitative real-time polymerase chain reaction (qRT-PCR) for the TSS of *claudin-5*, *VE-PTP*, and *vWf* performed on endogenous Ezh2-bound chromatin immunoprecipitated from confluent VEC-positive and VEC-null ECs expressing dominant negative form of Tcf4 (TCF4-DN) or GFP (green fluorescent protein; negative control). Inset: WB analysis of TCF4-DN and Ezh2 in extracts from confluent VEC-positive and VEC-null ECs expressing TCF4-DN or control GFP. Vinculin is the loading control. *P<0.05; **P<0.01, *t* test VEC-null GFP vs VEC-null TCF4-DN. IP indicates immunoprecipitation; and TL, total cell lysate.

We then investigated whether β-catenin could interact with PcG protein complex. Coimmunoprecipitation experiments showed an interaction between β-catenin and both Ezh2 and Suz12 (Figure 4A). Surprisingly, we detected a strong Ezh2/β-catenin association in VEC-positive confluent cells that was unlikely to be connected to the analyzed recruitment mechanism (Figure 4A, left). The meaning of this interaction will be further explored in the next section.

β-Catenin/FoxO1 association is known to stabilize FoxO1 binding to *claudin-5* promoter. When TCF4-DN (dominant negative form of Tcf4), lacking the β-catenin–interacting region, was overexpressed in VEC-null cells, β-catenin/DNA interaction was abrogated,^5^ and Ezh2 binding to *claudin-5*, *VE-PTP*, and *vWf* promoters was strongly weakened (Figure 4B), suggesting a stabilizing role for β-catenin on PcG protein association to target sites.

### VEC Associates With Ezh2 and Sequesters It at the Plasma Membrane

**Figure 5.**
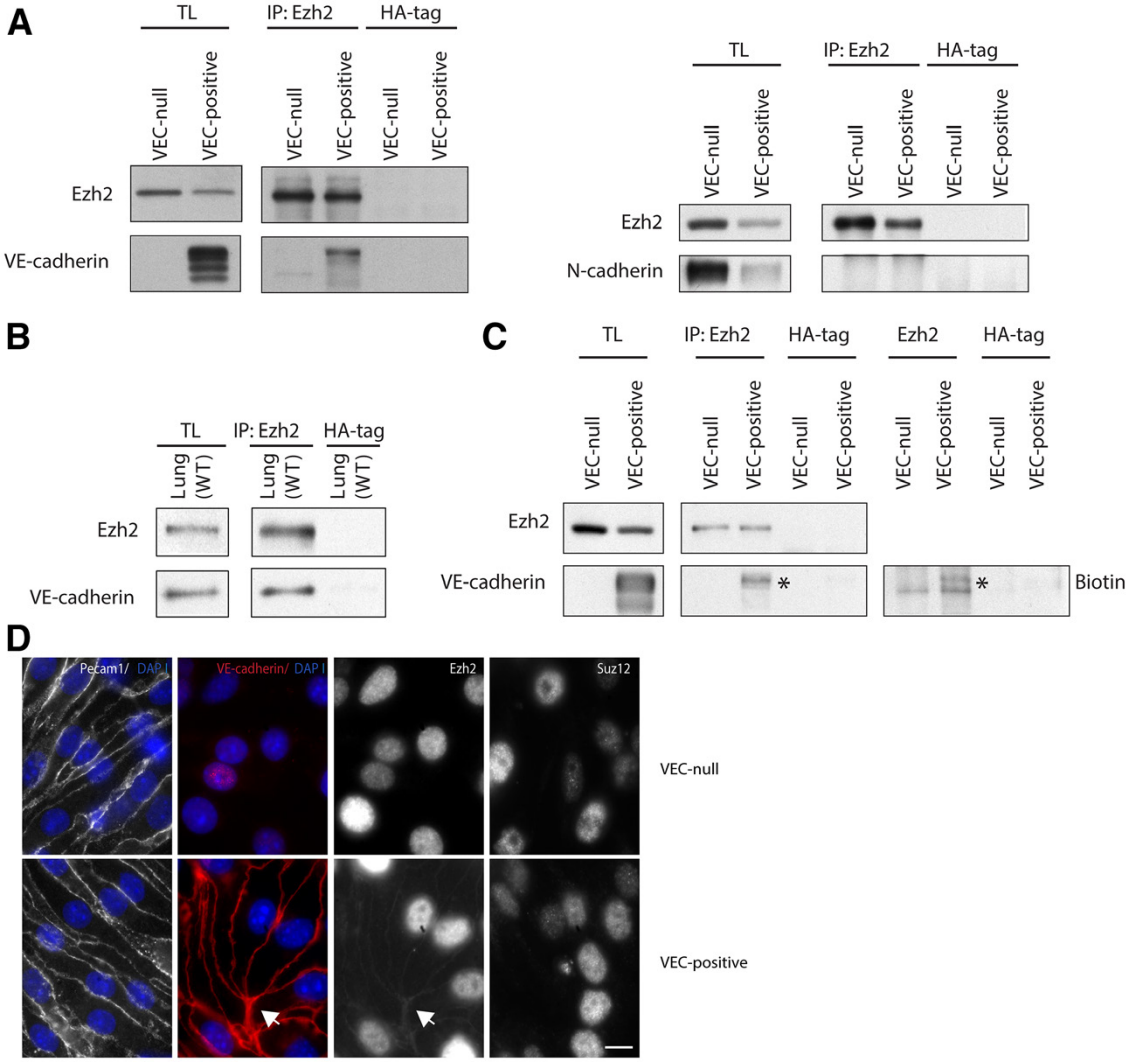
**VEC (vascular endothelial cadherin) sequesters Ezh2 (enhancer of zeste homolog 2) at the plasma membrane.** **A**, Coimmunoprecipitation and WB of endogenous Ezh2 and VEC or N-cadherin from extracts of confluent VEC-null and VEC-positive endothelial cells (ECs). **B**, Coimmunoprecipitation and Western blot (WB) of endogenous Ezh2 and VEC from wild-type (WT) murine whole lung extracts. **C**, Coimmunoprecipitation and WB of endogenous Ezh2 and VEC from extracts of confluent VEC-null and VEC-positive ECs after biotinylation of cell surface proteins. Asterisk highlights Ezh2-associated total and surface VEC bands. **D**, Immunofluorescence analysis of Ezh2 junctional localization (arrow) in confluent VEC-null and VEC-positive ECs. Junctional Suz (suppressor of zeste)12 was not detected. Platelet/endothelial cell adhesion molecule-1 (Pecam1) and VEC were used as junctional markers. Scale bar: 10 μm. IP indicates immunoprecipitation; and TL, total cell lysate.

The strong Ezh2/β-catenin association detected in confluent VEC-positive cells (Figure 4A, left) suggested an alternative role for such interaction in this cell type. Indeed, Ezh2 coimmunoprecipitated with endogenous full-length VEC (Figure 5A, left), whereas no interaction was detected between Ezh2 and N-cadherin (Figure 5A, right). Ezh2–VEC interaction was confirmed in vivo in adult mice-derived whole lung extracts (Figure 5B). Biotinylation of cell surface proteins further proved that Ezh2 associates with VEC exposed on the cell membrane (Figure 5C). Moreover, junctional staining of Ezh2 was detected in confluent VEC-positive but not in VEC-null ECs (Figure 5D, arrow). Taken together these data demonstrate that the observed VEC–Ezh2 interaction sequesters the polycomb protein at the cell surface.

**Figure 6.**
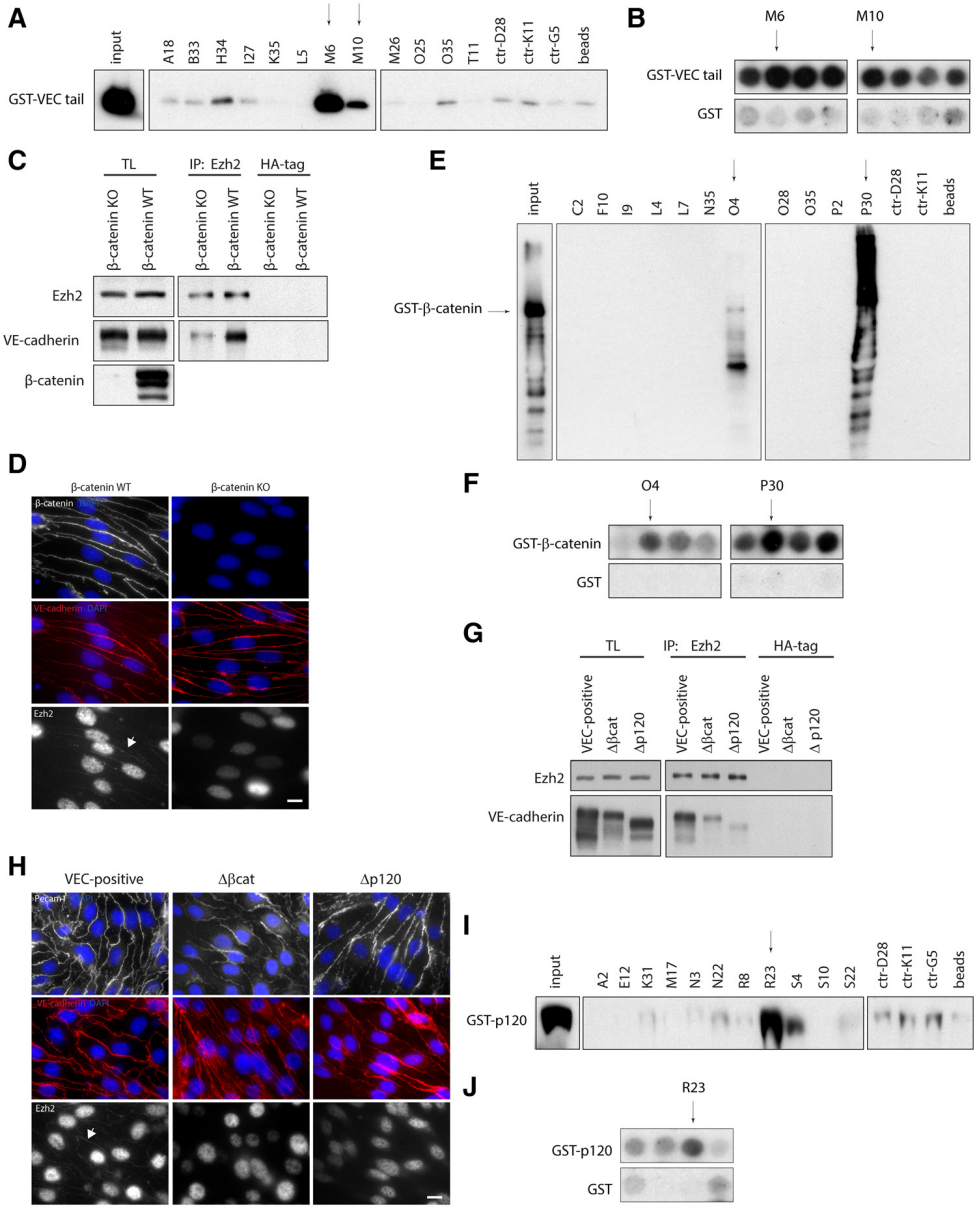
**Analysis of Ezh2 (enhancer of zeste homolog 2) interaction with VEC (vascular endothelial cadherin) junctional complex.** **A**, Streptavidin pull-down of selected biotinylated Ezh2 peptides and GST-tagged VEC cytoplasmic tail. GST-VEC cytoplasmic tail (400 ng) was loaded as input. Peptides displaying no interaction in peptide array were used as controls. Arrows indicate peptides showing positive signal. **B**, Regions of peptide array in the Online Figure IXA and IXB corresponding to selected peptides in (**A**). **C**, Coimmunoprecipitation and Western blot (WB) of Ezh2 and VEC from extracts of confluent β-catenin knockout (KO) and β-catenin wild-type (WT) endothelial cells (ECs). **D**, Immunofluorescence analysis of Ezh2 (arrow) and β-catenin junctional localization in confluent β-catenin KO and β-catenin WT ECs. VE-cadherin (red) was used as junctional marker. **E**, Streptavidin pull-down of selected biotinylated Ezh2 peptides and GST-tagged β-catenin. GST-β-catenin (400 ng) was loaded as input. Peptides displaying no interaction in peptide array were used as controls. Arrows indicate peptides showing positive signal. **F**, Regions of peptide array in the Online Figure IXA and IXC corresponding to selected peptides in (**E**). **G**, Coimmunoprecipitation and WB of Ezh2 and VEC from extracts of confluent VEC-positive, Δβcat, and Δp120 ECs. **H**, Immunofluorescence analysis of Ezh2 junctional localization (arrow) in confluent VEC-positive, Δβcat, and Δp120 ECs. VEC and platelet/endothelial cell adhesion molecule-1 (Pecam1) were used as junctional markers. **I**, Streptavidin pull-down of selected biotinylated Ezh2 peptides and GST-tagged p120-catenin. GST-p120-catenin (300 ng) was loaded as input. Peptides displaying no interaction in peptide array were used as controls. Arrow indicates peptide showing positive signal. **J**, Regions of peptide array in the Online Figure IXA and IXD corresponding to selected peptides in (**I**). In (**D**) and (**H**), scale bar: 10 μm. GST indicates glutathione *S*-transferase; HA, human influenza hemagglutinin; IP, immunoprecipitation; and TL, total cell lysate.

To define whether Ezh2 interacts directly with VEC cytoplasmic tail and which Ezh2 domain is involved in such interaction, we designed a peptide array displaying 20-mers covering the entire amino acid sequence of mouse Ezh2 (746 amino acids). Each peptide spotted on the membrane overlapped the next one by 19 amino acids, resulting in a change of only a single amino acid per peptide and providing a high resolution in determining the interaction sites. Such peptide array was probed with glutathione *S*-transferase (GST)–tagged VEC cytoplasmic tail^26^ and free GST as control for nonspecific interactions (Online Figure IXA and IXB). Peptides corresponding to the spots displaying high signal in VEC cytoplasmic tail–probed array and no or low signal in GST-probed control (Online Figure IXB, red dots) were synthesized as biotinylated and used in streptavidin pull-down experiments to confirm protein interactions (Figure 6A). Peptides M6 and M10 corresponding to amino acids 450 to 469 and 454 to 473, respectively, were identified as the sites of Ezh2/VEC direct interaction (Figure 6A and 6B).

We then investigated the role of VEC cytoplasmic partner β-catenin in such interaction. Ezh2/VEC association was reduced in a β-catenin–null EC line (β-catenin knockout) compared with its wild-type counterpart (β-catenin wild type; Figure 6C and 6D). When VEC-null cells were reconstituted with a truncated mutant of VEC lacking the β-catenin–binding domain (Δβcat),^5^ thus unable to sequester β-catenin at the membrane, VEC displayed a marked reduction of Ezh2 binding (Figure 6G and 6H). These results strongly suggested that β-catenin association to VEC cytoplasmic tail is required for optimal Ezh2 junctional recruitment. Ezh2 peptide array was probed with GST-tagged β-catenin (Online Figure IXC). Biotinylated peptide pull-down with peptides corresponding to the spots displaying high signal in β-catenin–probed array and no or low signal in GST-probed control (Online Figure IXC, red dots) confirmed Ezh2 interaction with β-catenin at amino acids 522 to 541 and 585 to 604, corresponding to peptides O4 and P30 (Figure 6E and 6F).

Ezh2 also associated with another constituent of VEC cytoplasmic junctional complex, p120-catenin (Online Figure X). VEC-null cells reconstituted with a mutant version of VEC lacking the juxtamembrane p120-catenin binding region showed a marked reduction of VEC/Ezh2 interaction (Figure 6G and 6H), suggesting a role for p120-catenin in such association. Ezh2 peptide array was probed with GST-tagged p120-catenin, and the identified putative interacting peptides (Online Figure IXD, red dots) were selected for validation by biotinylated peptide pull-down (Figure 6I). Amino acids 652 to 671, corresponding to peptide R23, were identified as Ezh2 interaction site with p120-catenin (Figure 6I and 6J).

Overall, these data prove that Ezh2 interacts with VEC junctional complex at multiple sites. Whether the identified interactions correspond to different junctional pools of Ezh2 or a single complex is formed is a matter of future investigation.

### Inhibition of Ezh2/VEC Interaction Downregulates Claudin-5, VE-PTP, and vWf Expression

**Figure 7.**
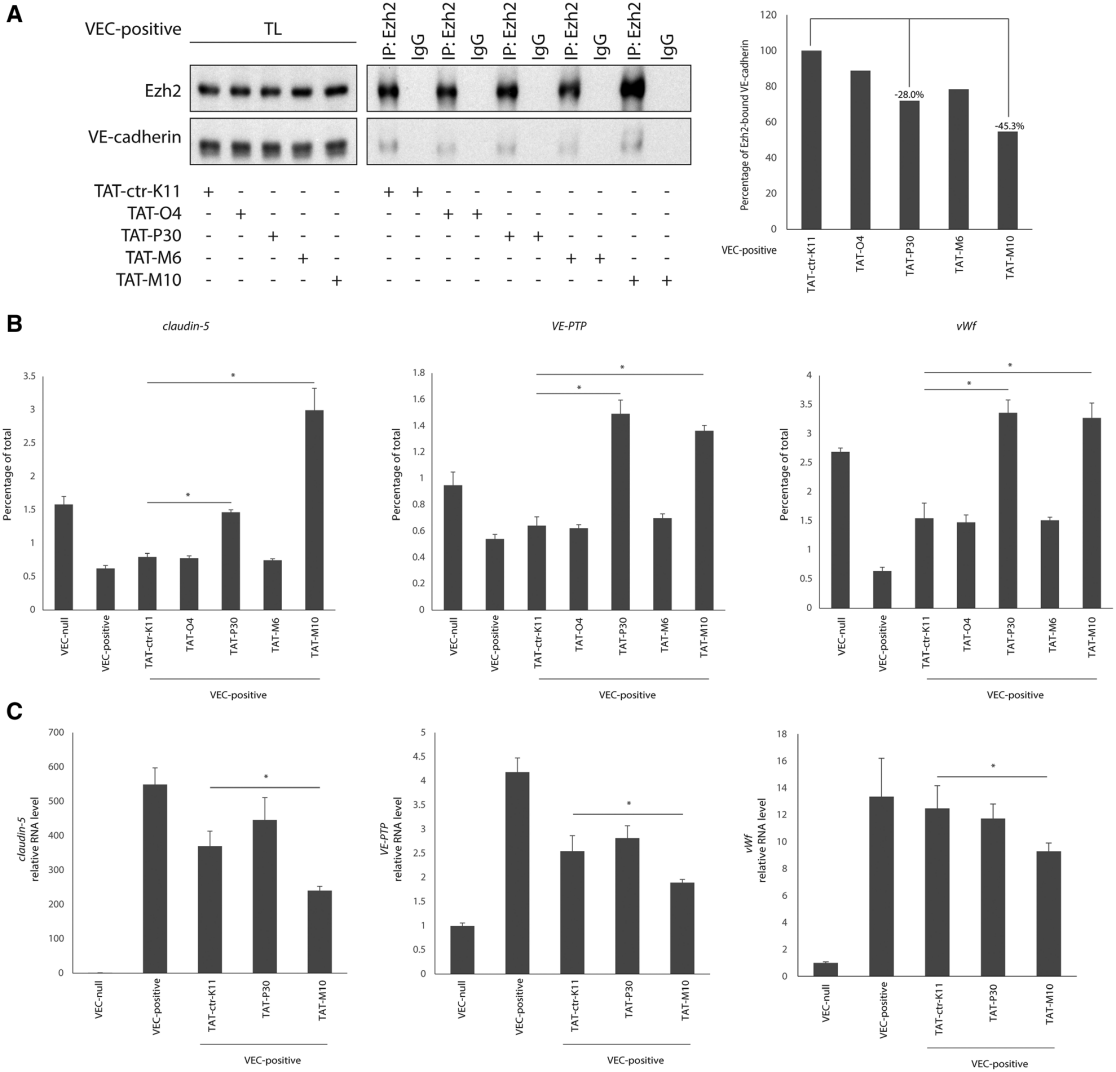
**Inhibition of Ezh2 (enhancer of zeste homolog 2)/VEC (vascular endothelial cadherin) interaction causes ***claudin-5***, vascular endothelial-protein tyrosine phosphatase (*VE-PTP*), and von Willebrand factor (*vWf*) downregulation.** **A**, Coimmunoprecipitation and Western blot (WB) of endogenous Ezh2 and VEC from extracts of VEC-positive endothelial cells (ECs) treated with VEC-binding transactivator of transcription (TAT)-M6 and TAT-M10 peptides, β-catenin–binding TAT-O4 and TAT-P30 peptides or nonbinding TAT-ctr-K11 peptide as control (**left**). Quantification of coprecipitated VEC protein normalized on precipitated Ezh2 level (**right**). **B**, Quantitative real-time polymerase chain reaction (qRT-PCR) for the transcription start site (TSS) of *claudin-5*, *VE-PTP*, and *vWf* performed on endogenous Ezh2-bound chromatin immunoprecipitated from VEC-positive ECs treated with VEC-binding TAT-M6 and TAT-M10 peptides, β-catenin–binding TAT-O4 and TAT-P30 peptides or nonbinding TAT-ctr-K11 peptide as control. Levels of DNA are normalized to input; columns are means±SD of triplicates from a representative experiment. *P<0.01, *t* test TAT-ctr-K11 vs TAT-P30 or TAT-M10 treatment. **C**, qRT-PCR analysis of *claudin-5*, *VE-PTP*, and *vWf* expression in VEC-positive ECs treated with VEC-binding TAT-M10 peptide, β-catenin–binding TAT-P30 peptide or nonbinding TAT-ctr-K11 peptide as control. Levels of mRNA are normalized to GAPDH; columns are means±SEM of triplicates from a representative experiment. *P<0.01, *t* test TAT-ctr-K11 vs. TAT-M10 treatment. IP indicates immunoprecipitation; and TL, total cell lysate.

To understand whether Ezh2 sequestration at AJs impacts the expression of *claudin-5*, *VE-PTP*, and *vWf*, we aimed at inhibiting Ezh2/VEC and Ezh2/β-catenin interactions. Selected Ezh2 peptides M6-M10 (VEC-binding sites) and O4-P30 (β-catenin–binding sites) were synthesized in tandem with transactivator of transcription (TAT) of human immunodeficiency virus, to allow peptide entry into the cell. Such TAT-conjugated peptides are expected to bind VEC or β-catenin engaging the domains needed for Ezh2 interaction, thus acting in a dominant negative fashion to inhibit Ezh2 junctional recruitment. VEC-positive cells were treated from subconfluent state throughout the period of time needed to establish AJs to saturate Ezh2-binding sites before the polycomb protein could localize at cell–cell contacts. Treatment with peptides TAT-P30 and TAT-M10 reduced VEC/Ezh2 association by 28% and 45.3%, respectively (Figure 7A). This caused an increase in Ezh2 recruitment to the TSS of *claudin-5*, *VE-PTP*, and *vWf* (Figure 7B), which, in the case of TAT-M10 treatment, corresponded to a significant repression of gene expression in comparison to treatment with a nonbinding TAT-control peptide (Figure 7C). The lack of gene downregulation after TAT-P30 treatment might be because of interference of this peptide with the correct assembly of the FoxO1/β-catenin/PRC2 complex at gene promoters.

### Polycomb Activity Correlates With Claudin-5, VE-PTP, and vWf Repression In Vivo

**Figure 8.**
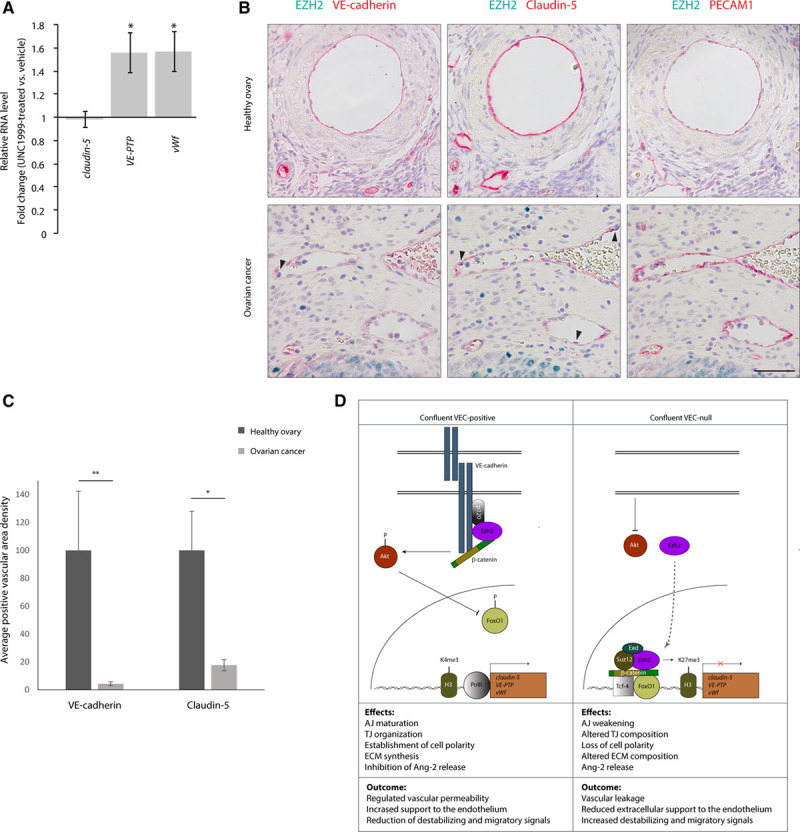
**Ezh2 (enhancer of zeste homolog 2) activity correlates with *claudin-5*, vascular endothelial-protein tyrosine phosphatase (*VE-PTP*), and von Willebrand factor (*vWf*) repression in vivo**. **A**, Quantitative real-time polymerase chain reaction (qRT-PCR) analysis of *claudin-5*, *VE-PTP*, and *vWf* expression in lungs of vehicle- or UNC1999-treated pups (P6). Data are represented as fold change of UNC1999 treated vs vehicle and are means±SD from at least 4 mice per group. Gene expression was normalized to *VEC* (vascular endothelial cadherin) expression. **B**, Immunohistochemistry (IHC) staining of VEC, Claudin-5, platelet/endothelial cell adhesion molecule-1 (PECAM1; red) and EZH2 (green) expression in serial sections of human healthy ovary (**upper**) or serous surface papillary ovarian carcinoma (**lower**). Black arrowheads point to tumor vessel endothelial cells (ECs) expressing high levels of EZH2. Scale bar: 50 μm. **C**, Quantification of IHC stainings in (**B**). For VEC and claudin-5, areas of specific signal, divided by the total measured area, were normalized to the corresponding values of PECAM1 staining. Columns are means±SEM (n=3 healthy ovaries; 4 ovarian carcinomas; at least 3 fields per sample). **D**, Suggested model for the regulation of *claudin-5*, *VE-PTP*, and *vWf* genes. Clustered VEC recruits β-catenin and activates Akt leading to FoxO1 (Forkhead box protein O1) phosphorylation and inhibition.^5^ Furthermore, Ezh2 is sequestered at the cell membrane by association with VEC cytoplasmic tail (**left**). These mechanisms allow gene activation by impeding the recruitment of PcG (polycomb group) proteins to gene promoters. Claudin-5 expression allows the correct organization of tight junctions (TJs) and regulation of vessel permeability.^5,27^ VE-PTP regulates adherens junction (AJ) maturation^28^ and VEGFR2 (vascular endothelial growth factor receptor-2) activity,^13,29^ whereas vWf contributes to extracellular matrix (ECM) formation and inhibits Ang (angiopoietin)-2 release.^14,30^ These effects are likely to contribute to vessel stabilization and prevent vascular leakage. In (**A**) and (**C**), *P<0.05; **P<0.01, *t* test.

To verify whether the inhibition of PRC2 activity could enhance the expression of the identified endothelial genes in vivo, we analyzed the effect of Ezh2/Ezh1 inhibition in mouse pups by pharmacological treatment with UNC1999. As reported in Figure 8A, drug administration increased *VE-PTP* and *vWf* expression in lung ECs, but was unable to increase *claudin-5* expression over physiological levels.

EZH2 overexpression is frequent in tumor cells and in vessels of human epithelial ovarian cancers and is associated with poor prognosis.^31^ Vessels of human healthy ovarian tissue presented very low EZH2 expression, while VEC, Claudin-5, and PECAM1 were clearly detectable (Figure 8B, upper). In contrast, in tumor vasculature, high nuclear EZH2 staining paralleled a significant reduction in VEC and Claudin-5, whereas PECAM1 expression was unaltered (Figure 8B, lower, and 8C). EC-associated vWf staining was highly variable in both healthy ovarian tissue and tumor samples, preventing a quantitative evaluation of its expression. VE-PTP staining quantification also presented problems of antibody specificity. Nonetheless, a reduction of VE-PTP signal could be appreciated in tumor vessels (Online Figure XI).

## Discussion

In this study, we describe a novel mechanism through which VEC expression and clustering upregulate endothelial-specific genes and contribute to endothelial differentiation and stability. We found that VEC engagement at cell–cell contacts acts by inhibiting PcG protein–mediated epigenetic modifications of EC chromatin. PcG proteins have been implicated in several developmental processes,^7,8^ and Ezh2 activity was claimed to be involved in the regulation of genes important in tumor angiogenesis and Kaposi Sarcoma.^31^ Furthermore, Ezh2 was reported to play a role in maintaining vascular integrity during embryonic development.^32^

However, these studies mainly focused on the effects of Ezh2 inhibition without investigating in detail the mechanisms of polycomb regulation in ECs. We observed that VEC expression and clustering were able to trigger a gene expression program contributing to endothelial differentiation. Some of these endothelial genes seemed to be regulated by polycomb activity, thus pointing to a possible central role of VEC/polycomb crosstalk in orchestrating endothelial specification.

Within the set of genes upregulated by VEC, we selected endothelial-specific *claudin-5*, *VE-PTP*, and *vWf*. Claudin-5 is a major component of endothelial TJs and determines size and charge selectivity of endothelial paracellular permeability.^4,5,27,33^

VE-PTP exerts different context-specific activities including associating with VEC and reducing its tyrosine phosphorylation.^34^ Absence of VE-PTP impairs AJ maturation, inducing vessel destabilization and increased permeability.^28,35^ VE-PTP associates with Tie-2 receptor modulating Ang (angiopoietin) signaling^36^ and interacts with VEGFR2 (vascular endothelial growth factor receptor-2) limiting its signaling activity.^13,29^

vWf is a crucial component of EC extracellular matrix that provides mechanical support to the EC monolayer^30^ and limits the release of vessel-destabilizing Ang-2 from endothelial Weibel–Palade bodies.^14^ Consistent with our data, a larger pool of vWf is present in confluent ECs compared with subconfluent condition.^37^

Although our analysis was mainly focused on these 3 genes, other genes regulated by VEC are involved in inhibition of cell proliferation, decrease of sprouting angiogenesis, promotion of cell adhesion, and production of extracellular matrix, suggesting a broader role of VEC expression and clustering in inducing vessel stabilization.

We previously found^5^ that VEC expression derepresses the endothelial-specific gene *claudin-5* by restraining the activity of FoxO1 and β-catenin.^33^ Here, we show that nuclear FoxO1 and β-catenin increase polycomb targeting to the promoter regions of a set of endothelial genes. We observed that induction of *claudin-5*,^5^ *VE-PTP*, and *vWf* relies on VEC capacity to activate the PI3K/AKT pathway, leading to FoxO1 inactivation,^4^ and to sequester β-catenin at the cell membrane. The absence of VEC clustering prevented the expression of this set of genes. The work presented here confirms and extends what previously observed for *claudin-5*^5^ and further underlines the role of FoxO1 as a key transcription factor in endothelial differentiation and homeostasis.^38^ This is in agreement with a recent report highlighting a key role of Akt1 activation and FoxO inhibition in stabilizing the endothelial barrier and preventing vascular leakage.^39^

Little is known about the molecular mechanisms of FoxO1-mediated gene repression. We found that FoxO1 physically associates with PcG proteins and, through still unknown mechanisms, contributes to their association to a selected set of endothelial gene promoters inducing chromatin conformational changes and gene inhibition. β-Catenin takes part in such multiprotein complex and stabilizes PcG protein binding to DNA, as TCF4-DN–mediated abrogation of β-catenin/DNA binding destabilizes Ezh2/promoter interaction.

PcG proteins have been previously reported to have atypical extranuclear localizations.^40,41^ We show here that VEC also reduces polycomb nuclear activity by sequestering Ezh2 at the plasma membrane in a β-catenin–dependent and p-120-catenin–dependent manner. Ezh2 interaction with VEC was confirmed in vivo in mouse lung extracts in the absence of any crosslinking reaction, whereas no association was detected between Ezh2 and the other major classical endothelial cadherin, N-cadherin, likely as a result of the reduced binding of this adhesion molecule to p120-catenin.^42^ Recently, EZH2 has been shown to interact with β-catenin in liver cancer stem cells^43^ through its N-terminal domain (amino acids 1–334). This was defined by domain mapping assays using EZH2 truncation mutants. Our analysis identified the Ezh2 sites of direct interaction with β-catenin in the domain comprised between amino acids 522 and 604. Deletion of domain 1 to 334 might alter the protein structural integrity, affecting EZH2/β-catenin interaction at downstream residues.

Ezh2/VEC association was shown to play a functional role in regulating gene expression. Inhibition of such interaction, and likely the consequent release of Ezh2 protein in the cytoplasm, increased its recruitment to *claudin-5*, *VE-PTP*, and *vWf* gene promoters causing their downregulation. The strong increase of Ezh2 recruitment at gene promoters, however, did not parallel a similarly strong repression of gene expression.

This was likely because of reduced FoxO1 and β-catenin nuclear abundancy in the confluent VEC-positive condition, impeding the correct assembly of the nuclear repressive complex.

Thus, we propose that an event happening at cell–cell contacts, that is, VEC clustering, triggers and orchestrates a network of pathways and epigenetic events, regulating several aspects of endothelial homeostasis and vascular stability (Figure 8D).

Pharmacological inhibition of Ezh2/Ezh1 led to the upregulation of *VE-PTP* and *vWf* in mouse pups, confirming that these genes are dependent on polycomb activity in vivo. C*laudin-5* was not significantly upregulated by such treatment, likely because its expression is unable to increase over a physiological threshold. Additional studies are needed for understanding the kinetics of expression of this gene in the presence or absence of polycombs in different vascular regions.

Deregulated angiogenesis is a characteristic of several human pathologies and is a hallmark of cancer. Tumor vessels present altered endothelial cell–cell junctions, are hyperpermeable, have low pericyte coverage, and altered basement membrane composition and stiffness. Tumor vessel stabilization by increased VEC has been shown to reduce tumor metastasis.^44,45^ Ezh2 expression is increased in tumor-associated ECs, and this has been linked to increased vascular density and reduced vessel maturation.^31^ Our results show that reduced VEC and increased EZH2 nuclear staining in human ovarian carcinoma vessels parallel the downregulation of Claudin-5 and VE-PTP, suggesting a possible alteration of the identified signaling mechanism.

In conclusion, this work introduces a novel role for VEC in the regulation of polycomb activity and, consequently, EC differentiation and vascular maturation. The available genetic and pharmacological approaches to inhibit polycomb function will be instrumental to identify the role of this mechanism in different pathological conditions of the vascular system and to investigate new therapeutic opportunities to induce vessel normalization in cancer.^4^

## Acknowledgments

We thank Luca Ferrarini for help in bioinformatic analysis, Djamil A. Damry for scientific input, and Emanuele Martini and Dario Parazzoli for imaging analysis. The anti–vascular endothelial-protein tyrosine phosphatase (VE-PTP; hPTPb1-8) antibody and FKHR-TM (Forkhead transcription factor triple mutant) adenovirus were kind gifts of Dietmar Vestweber and Christopher Daly, respectively.

## Sources of Funding

This work was supported by the Francis Crick Institute which receives its core funding from Cancer Research UK (FC001057), the UK Medical Research Council (FC001057), and the Wellcome Trust (FC001057); by Associazione Italiana per la Ricerca sul Cancro (AIRC; investigator grant [IG] 16683), AIRC IG2016 18683 and Special Program Molecular Clinical Oncology 5x1000 to AGIMM (AIRC Gruppo Italiano Malattie Mieloproliferative; 10005); by the European Research Council (project EC-ERC-VEPC contract 742922); by ITN (initial training networks) BtRAIN grant 675619; by CARIPLO Foundation (2008.2463) and TELETHON (GGP14149). Research was also supported by the grants R01CA218600, R01GM122749, and R01HD088626 (to J. Jin) from the US National Institutes of Health. Sequencing was performed by the SNP&SEQ Technology platform in Uppsala. The facility is part of the National Genomics Infrastructure (NGI) Sweden and Science for Life Laboratory. The SNP&SEQ Platform is also supported by the Swedish Research Council and the Knut and Alice Wallenberg Foundation. E. Dejana is supported by the Swedish Science Council and the Knut and Alice Wallenberg Foundation.

## Disclosures

None.

## Supplementary Material

## Novelty and Significance

### What Is Known?

- Pathological conditions such as inflammation, diabetic retinopathy, and age-related macular degeneration are characterized by deregulated angiogenesis and unstable blood vessels.
- Solid tumors receive nutrients and oxygen from newly formed vessels that display structural abnormalities such as lack of hierarchy, abnormal lumen, altered endothelial cell–cell junctions, and poor control of permeability.
- VEC (vascular endothelial cadherin) and its clustering at cell–cell adherens junctions upregulate claudin-5, key component of endothelial tight junctions regulating vessel permeability, via inhibition of FoxO1 (Forkhead box protein O1) and β-catenin nuclear translocation.
