# Supplementary material for: VE-Cadherin–Mediated Epigenetic Regulation of Endothelial Gene Expression
Source: Circ Res. 2017 Dec 12;122(2):231–45. doi: 10.1161/CIRCRESAHA.117.312392 (PMC5771688; doi:10.1161/CIRCRESAHA.117.312392)
Supplement: Supplementary file 1 [file res-122-231-s001.pdf]

SUPPLEMENTAL MATERIAL

VE-cadherin-mediated Epigenetic Regulation of Endothelial Gene Expression

M. F. Morini, C. Giampietro, M. Corada, F. Pisati, E. Lavarone, S. I. Cunha, L. L. Conze, N. O'Reilly, D. Joshi, S. Kjaer, R. George, E. Nye, A. Ma, J. Jin, R. Mitter, M. Lupia, U. Cavallaro, D. Pasini, D. P. Calado, E. Dejana and A. Taddei

DETAILED METHODS

Data Disclosure
The data that support the findings of this study are available from the corresponding author upon reasonable request.

Cell culture
The cell lines used in this study were:
- ECs derived from murine embryonic stem cells with homozygous null mutation of the *VEC* gene (VEC-null)[1]. The wild type form of *VEC* was introduced in these cells (VEC-positive) using retrovirus-mediated transfer as described in detail by Lampugnani et al.[2];
- Δβcat cells, ECs expressing a truncated mutant of VEC (lacking residues 703-784 of human VEC)[3], which correspond to the β-catenin-binding region[2];
- Δp120 cells, ECs expressing a truncated mutant of VEC (lacking residues 621-702 of human VEC), which correspond to the p120-catenin-binding region[2];
- IL2-VEC cells, ECs expressing a mutated version of VEC made up of the VEC cytoplasmic domain (amino acids 621-784) fused to the extracellular and transmembrane domains of IL-2 receptor α-chain (from Andrew Kowalczyk, Emory University, Atlanta, GA)[4, 5];
- β-Catenin WT and β-catenin KO ECs were derived from lungs of adult β-catenin$^{flox/flox}$ mice[6], immortalized as previously described[1, 7] and infected with an adenovirus encoding GFP (control) or CRE recombinase to obtain *β-catenin* gene recombination.

For all ECs of murine origin, culture medium was DMEM (GIBCO) with 20% North American (NA) fetal bovine serum (FBS) (HyClone), glutamine (2 mM; Sigma), penicillin/streptomycin (100 units/l; Sigma), sodium pyruvate (1 mM; Sigma), heparin (100 µg/ml, from porcine intestinal mucosa; Sigma), and EC growth supplement (ECGS) (5 µg/ml, made in our lab from calf brain) (complete culture medium). Starving medium was MCDB 131 (GIBCO) with 1% bovine serum albumin (BSA) (EuroClone), glutamine (2 mM), penicillin/streptomycin (100 units/l) and sodium pyruvate (1mM).
- Human dermal microvascular endothelial cells-1 (HMEC-1) (from STP Cell Services, The Francis Crick Institute, London, UK) were cultured in MCDB 131 (Thermo Fisher) with 10% North American (NA) fetal bovine serum (FBS) (HyClone), glutamine (10 nM; Sigma), penicillin/streptomycin (100 units/l; Sigma), epidermal growth factor (EGF) (30ng/ml; Sigma) and hydrocortisone (1 µg/ml; Sigma);
- 293T-Phoenix-Ecotropic packaging cells were provided by IFOM Cell Culture facility and cultured in DMEM medium supplemented with 10 % South American (SA) FBS (Hyclone), glutamine (2 mM) and sodium pyruvate (1 mM);
- Low passage AD-HEK293 cell line (human embryonic kidney, American Type Culture Collection, Manassas, VA), used for adenoviral production, were provided by IFOM Cell Culture facility and grown in DMEM medium supplemented with 10% FBS NA, glutamine (4 mM), penicillin/streptomycin (100 units/l), and sodium pyruvate (1 mM).

All cells were cultured at 37°C in a humidified atmosphere with 5% CO_2.

Lentiviral and adenoviral preparations
ΔN-βcatenin construct was obtained from C. Brancolini (University of Udine, Udine, Italy). Lentiviral vectors used to stably express a short hairpin RNA against Suz12 (sh-Suz12), to overexpress Suz12 and the respective controls were a kind gift of D. Pasini (IEO, Milan, Italy). Packaging plasmids were kindly donated by L. Naldini (HSR-TIGET, San Raffaele Telethon Institute for Gene Therapy, Milan, Italy).

Lentiviral vectors were produced as described by Dull et al.[8]. Briefly, on day 1, 293T-Phoenix-Ecotropic packaging cells were transfected with the viral genome using calcium phosphate and incubated overnight with the transfection mix. On day 2 the medium containing the transfection mix was removed and 293T-Phoenix-Ecotropic cells were grown in as little medium as possible to concentrate the virus. On day 3 the medium containing the virus was removed, passed through a 0.45 μm diameter filter, supplemented with Polybrene (8 μg/ml, from IFOM Cell Culture facility) and placed on cells to be infected. The same procedure was repeated on day 4. Sh-Suz12- and sh-Empty-infected cells were selected with hygromycin 300 μg/ml. Cells were kept under selection until control non-infected cells died. Suz12-overexpressing cells and their Empty control were selected with Puromycin 3 μg/ml. Cells were kept under selection until control non-infected cells died.
The FKHR-TM adenovirus has been previously described[9]. The TCF4-DN adenovirus was kindly donated by S. J. George (Bristol Heart Institute, Bristol, UK)[10]. Infectious viruses were purified and titered using standard techniques. Briefly, for adenovirus production AD-293T cells were infected with 2 pfu/cell in DMEM without serum for 1 h at 37°C. Then, the infection medium was removed and cells were grown in an appropriate volume of DMEM + 5% horse serum until complete cell lysis was obtained (usually 72h later). The medium containing the viruses was then subjected to 3 freeze-and-thaw cycles in order to destroy all the cells and to free as many virions as possible. The resulting supernatant was then centrifuged at 3000 rpm for 30 min at 4°C to eliminate the cellular debris, aliquoted and stored at -80°C. For the infection of ECs two consecutive cycles of infection [5 h and overnight (O/N)] were performed with MOI of 300 in 1 ml of complete culture medium.

Immunofluorescence microscopy
Immunofluorescence microscopy staining was performed using standard technique, as previously described[11]. Briefly, cells were seeded on 0.5% cross-linked gelatin. Cells were fixed and permeabilized in ice-cold methanol at -20°C for 5 min. Fixed cells were incubated for 30 min in a blocking solution [phosphate buffer saline (PBS) containing Ca^{2+} and Mg^{2+}, 2.5% skim-milk, 0.3% TritonX-100]. Cells were then incubated for 1 h at RT with primary antibodies diluted in blocking buffer. Alternatively, cells were fixed with 1% PAF in triethanolamine, pH 7.5, containing 0.1% Triton X-100 and 0.1% NP-40 for 20 min. Fixed cells were incubated for 30 min in a blocking solution [Tris-buffered saline (TBS) containing 5% BSA, 0.3%TritonX-100]. Cells were then incubated overnight at 4°C with primary antibodies diluted in blocking buffer. Appropriate secondary antibodies were applied on cells for 45 min at RT and cells were then mounted with VECTASHIELD containing DAPI (Vector Biolabs).
Samples were observed under an epifluorescence microscope (DMR; Leica) using a 63X objective. Images were captured using a charge-coupled camera and processed with Adobe Photoshop. Only adjustments of brightness and contrast were used in the preparation of the figures. For comparison purposes, different sample images of the same antigen were acquired under constant acquisition settings.

Western blot analysis
Total proteins were extracted by solubilising cells in boiling Laemmli buffer [2.5% SDS and 0.125 M Tris-HCl (pH 6.8)]. Lysates were incubated for 5 min at 100°C to allow protein denaturation and then spinned for 5 min at 13200 rpm to discard cell debris. The supernatants were collected and the concentration of protein was determined using a BCA™ Protein Assay Kit (Pierce) according to manufacturer's instructions. Equal amounts of proteins were loaded on gel and separated by SDS-PAGE, transferred to a Protran Nitrocellulose Hybridization Transfer Membrane 0.2 μm pore size (Whatman) and blocked for 1 h at RT in 1X TBS Tween (TBST) [150 mM NaCl, 10 mM Tris-HCl (pH 7.4), and 0.05% Tween] containing 5% (w/v) skim-milk. The membranes were incubated overnight at 4°C or 1 h at RT with primary antibodies diluted in 1X TBST-5% BSA. Next, membranes were rinsed 3 times with 1X TBST for 5 min each and incubated for 1 h at RT with HRP-linked secondary antibodies (diluted in 1X TBST-5% BSA). Membranes were rinsed 3 times with TBST for 5 min each and specific binding was detected by the enhanced chemiluminescence (ECL) system (Amersham Biosciences) using HyperfilmTM (Amersham Biosciences) or the ChemiDoc gel imaging system (BIORAD). The molecular masses of proteins were estimated relatively to the electrophoretic mobility of co-transferred prestained protein marker, Broad Range (Cell Signalling Technology).

Co-immunoprecipitation
Cells were grown until confluent and starved overnight. Cells were then washed once with DMEM without serum and incubated with 0.4 mg/ml of dithiobis(succinimidyl)propionate (DSP) (Pierce) for 30 min at 37°C. After several washes with ice-cold PBS, cells were lysed in ice-cold modified RadioImmunoPrecipitation Assay (RIPA) buffer (Tris HCl pH 7.5 100 mM, NaCl 150 mM, Deoxycholic acid 1%, SDS 0.1%, $CaCl_2$ 2

mM). The protein lysate was precleared with an appropriate volume of Protein G Sepharose 4B (Zymed) for 3 h at 4°C. Protein concentration was determined with BCA™ Protein Assay Kit and an equal amount of protein was incubated with either immune antibodies or species-matched control antibodies overnight at 4°C. On the following day immunocomplexes were collected using Protein G Sepharose 4B for 3 h at +4°C. Beads were then washed several times with modified RIPA buffer and boiled in an appropriate volume of Laemmli buffer. Samples were analysed by standard Western blot analysis as described above[11].

CoIP following biotinylation of membrane proteins was performed using the same protocol as above. Before cell lysis with modified RIPA buffer cells were incubated with Sulfo-NHS-LC-Biotin (Pierce ThermoScientific) 0.55 mg/ml in PBS containing Ca^{2+} and Mg^{2+} pH 8.0 for 30 min at 4°C. After biotinylation cells were washed with PBS containing Ca^{2+} and Mg^{2+}pH 8.0 + Glycine 100 mM to quench the reaction.

CoIP from lung tissue: lungs from wild type adult age-matched mice were lysed in ice-cold modified JS buffer [Hepes pH 7.5 72 mM, NaCl 210 mM, glycerol 0.5 %, Triton X-100 1%, $MgCl_2$ 2 mM, EGTA 7.2 mM, SDS 0.1 %, Sodium Orthovanadate 300 mM, Pefabloc SC 1 mM (Sigma) and Sodium Fluoride 1 mM] using Tissue Lyser II (QIAGEN) (two 30-sec pulses at maximum frequency). Samples were precleared for 4 h at 4°C with an appropriate volume of Protein G Sepharose 4B. Protein concentration was determined with BCA™ Protein Assay Kit and an equal amount of protein was incubated with Protein G Sepharose 4B pre-coupled with either immune antibodies or species-matched control antibodies overnight at 4°C. The day after, beads were then washed with modified JS buffer and boiled in an appropriate volume of Laemmli buffer. Samples were analysed by standard western blot analysis[11]. All the buffers contained freshly added protease inhibitor cocktail (IFOM Kitchen Facility).

RNA-seq library preparation and sequencing

1 µg total RNA per sample (in triplicate) was used to prepare RNA sequencing libraries of VEC-null and VEC-positive cells with SMARTer Stranded Total RNA Sample Prep kit - High input Mammalian kit (Clontech), according to the manufacturer's instructions. This kit includes depletion of ribosomal RNA prior to library synthesis, barcoding and amplification. For validation of the amplified RNA-seq library, 1 µl of the library was used with the Agilent High Sensitivity DNA kit (Agilent). The samples were sequenced on a HiSeq2500 sequencing system with v4 chemistry from Illumina at the Swedish National Genomics Infrastructure, Uppsala node, SNP&SEQ technology platform (Science for Life Laboratory, Uppsala). The samples were run in pools of 6 samples with equimolar amounts of dsDNA from each of the six samples. Each pool was run on a single lane (paired-end sequencing, 125bp reads).

Bioinformatics analysis of RNA-seq data

Each sample received ~24 to 46 million of paired-end reads. Trim Galore (http://www.bioinformatics.babraham.ac.uk/projects/trim_galore/) was used for quality/adapter trimming of the raw reads. In addition, 5 nucleotides from both 5' and 3' end of the reads were clipped to remove unwanted bias. Then trimmed reads were mapped to the mm10 mouse genome with > 70% overall mapping rate using TopHat v2.1.1[12] and annotated with GO terms using R package Biomart[13]. Differential gene expression (DEG) analysis was performed using DESeq2[14]. Genes were considered significantly differentially expressed if the $|\log2FC| \geq 1$ and the adjusted p-value ≤ 0.05.

Identification of enriched functional annotation categories

Genes significantly up and downregulated in confluent VEC-positive vs. VEC-null cells were used to query DAVID's[15] "Functional Annotation Clustering" tool to look for enrichment of annotation terms from GOTERM_BP_DIRECT, KEGG, BIOCARTA and UP_KEYWORDS using the *Mus musculus* genome as background. Annotation terms with overlapping gene sets were grouped (see Online Table III for details) and used to annotate an expression heat-map of the same genes across all samples. For the purposes of visualisation abundance is presented as log2 (normalised read count + 1).

Quantitative Real Time (qRT)-PCR analysis

Total RNA was isolated by RNeasy kit (QIAGEN) and 1 µg was reverse transcribed with random hexamers (High Capacity cDNA Archive Kit, Applied Biosystems) according to the manufacturer's instructions. cDNA (5 ng) was amplified in triplicate in a reaction volume of 15 µl using TaqMan Gene Expression Assay (Applied Biosystems) and an ABI/Prism 7900 HT thermocycler, using a pre-PCR step of 10 min at 95°C, followed by 40 cycles of 15 sec at 95°C and 60 sec at 60°C. Preparations of RNA template without reverse transcriptase were used as negative controls. For any sample the expression level, normalized to the housekeeping gene

18S, glyceraldehyde-3-phosphate dehydrogenase (GAPDH) or β2-microglobulin, was determined using the comparative threshold cycle (CT) method as previously described[16].

Transcription factor binding site analysis

In order to identify FoxO1 and Tcf/β-catenin consensus sequences on the putative *VE-PTP* and *vWf* promoter regions we used the program MatInspector[17], which identifies transcription factor binding sites (TFBS) in nucleotide sequences using a large library of weight matrices. We analyzed the sequence spanning from 6000 bp upstream and 500 bp downstream the transcription start site (TSS) of the genes and obtained a prediction of a potential combination of TFBS. The TFBS sequences considered in the analysis were [AG][GA][TG][AC]AACAA[AC] for FoxO1 binding and [TAG][GT][AG][CT][AT]x(2)CAAAG[GCT][GAC][AC][GCA] for Tcf/β-catenin binding.

***Claudin-5* promoter luciferase assay**

Claudin-5 putative promoter region including all three regions of paired Tcf/β-catenin-FoxO1 binding sites[18] (3169 bp upstream of the CDS) was cloned into the XhoI-HindIII site of the multiple cloning region of pGL3-Basic Vector (Promega) using the following primers: 5'-AAAAACTCGAGAAATGGCTCTGGGCAAGAAG-3' and 5'-AGGGAAAGCTTGGCTAAAGACTGAATGCTCA-3'. XhoI and HindIII restriction enzymes were from New England BioLabs. Cloning was confirmed by sequencing, performed by STP Genomics Equipment Park (The Francis Crick Institute, London, UK). VEC-positive cells, wild type or infected with the indicated constructs, were seeded at 30.000 cells/cm^2; VEC-null cells, wild type or infected with the indicated constructs, were seeded at 55.000 cells/cm^2 in 35 mm diameter plates (Corning) in complete culture medium without penicillin/streptomycin. The day after, cells were transiently transfected with 2 µg/well of *Claudin-5*-pGL3-luciferase vector and 1 µg/well of pRL-TK vector (Promega) using 8 µl/well of LipofectAMINE 2000 (Invitrogen) in OptiMEM (Invitrogen) according to the manufacturer's instructions. After 8.5 h of incubation, cells were incubated in fresh complete culture medium and 72 h after transfection, firefly and Renilla luminescence were detected using Dual-Glo Luciferase Assay System (Promega), according to the manufacturer's instructions, with EnVision 2102 MultiLabel Reader (PerkinElmer). Renilla luminescence signal was used as normalizer for transfection efficiency.

Protein expression and purification

For GST-tagged VEC-cytoplasmic tail production, bacterial expression vector[19] was transformed into BL21 strain of *E. coli*. For growth, a single colony was picked from freshly transformed plate and allowed to grow overnight at 37°C in 20 ml LB medium. This culture was used to inoculate 2 x 1 liter of LB and TB media. Cultures were allowed to grow until the OD600nm reached 0.6 at which point protein expression was induced by the addition of 1 mM IPTG. Induction was allowed to occur at 37°C for 4 h. The cells were then harvested by centrifugation and stored at -80°C until required. Cells obtained from 1 liter of LB or TB media were treated in the same way. Frozen pellets were thawed and resuspended in 20 ml ice cold lysis buffer containing 50 mM HEPES (pH 7.5), 2 mM EDTA, 1 mM DTT and protease inhibitors (Sigma) (2 protease inhibitor tablets were added to 50 ml total lysis buffer). To ensure complete lysis the suspension was sonicated for 5 x 30 sec, 20% amplitude. The insoluble fraction was removed by centrifugation (30,000 rpm, 20 mins, 4°C) and the soluble fraction incubated with 200 µl glutathione sepharose for 1 h at 4°C with constant rolling. The resin was then washed extensively with lysis buffer and bound protein eluted using lysis buffer containing 20 mM reduced glutathione. Elutions were concentrated to a volume of 270 µl to which 30 µl of glycerol was added. The protein concentration was determined by Bradford reagent, snap frozen in liquid nitrogen and stored at -80 °C until required.

For GST-tagged p120-catenin production, competent *E.coli* DH10 Bac cells (Invitrogen) were transformed with GST-Ctnnd1-pFastBAC1 plasmid (NovoPro). White colonies were picked from freshly transformed plates and grown in liquid culture in order to purify bacmid DNA. This DNA was then used to generate virus by transfecting Sf21 insect cells (Invitrogen). Two further rounds of amplification generated high titre virus stocks suitable for infecting large scale cultures for expression of the protein. Sf21 insect cells were infected at a cell density of 1.2 x 10^6 cells/ml and at a MOI of 1. Infected cultures were allowed to grow for 3 days with shaking (140 rpm) at 27°C. Cells were then harvested and cell pellets stored at -80 ^{o}C until required. 2L of cell pellet was thawed and resuspended in 30 ml lysis buffer (50 mM HEPES [pH 7.5], 250 mM NaCl, 10% glycerol, 1 % Triton X-100, 1 mM NaF, 10 mM benzamidine, 0.5 mM EDTA, 10 mM B-glycerophosphate, 1 mM Na_2VO_4, 1mM DTT) and lysed by sonication on ice (3 min total time, 20 sec on 5 sec off, 20%

amplitude). Insoluble material was removed by centrifugation at 30.000 rpm for 20 mins. The soluble fraction was incubated with 300 µl bed volume of glutathione sepharose resin for 2 h (with rolling at 4°C) after which time the resin was washed extensively in buffer containing 50 mM HEPES (pH7.5), 250 mM NaCl, 1 mM DTT. Protein was eluted in the same wash buffer supplemented with 15 mM reduced glutathione and 5% glycerol.

Peptide arrays

Peptide arrays were synthesised on an Intavis Multipep Peptide Synthesiser (Intavis Bioanalytical Instruments AG), using N-Fmoc amino acids, onto a cellulose sheet derivitised to have 8-10 ethylene glycol units between the sheet and an amino group for synthesis. Each amino acid was coupled as a chlorohydroxybenzatriazole active ester, automatically formed immediately prior to use. Once the required number of cycles of coupling and deprotection and washing were completed, the membranes were treated with a solution of 20 mls containing 95% trifluoroacetic acid, 3% triisopropylsilane and 2% water for 4 h. Following this treatment, membranes were washed 4 times with dichloromethane, 4 times with ethanol, twice with water, once with ethanol.

Membranes were activated with 100% methanol for 10 seconds and washed twice with 1X TBST (0.1% Tween) for 5 minutes each wash. Membranes were then blocked for 1 h at RT in 1X TBST (0.1% Tween) containing 5% (w/v) skim-milk, and incubated 2 h at RT with free GST (kind gift of S. Tooze, The Francis Crick Institute, London, UK) or GST-tagged VEC-cytoplasmic tail[19], β-catenin (Sino Biological Inc.) or p120-catenin, diluted to 1 µg/ml in 1X TBST-5% BSA. Next, membranes were rinsed 5 times with 1X TBST (0.1% Tween) and incubated overnight at 4°C with HRP-linked anti-GST antibody (diluted in 1X TBST-5% skim-milk). Membranes were rinsed 5 times with 1X TBST (0.1% Tween) for 5 min each time and specific binding was detected by the enhanced chemiluminescence (ECL) system (Amersham Biosciences) using HyperfilmTM (Amersham Biosciences).

Peptide pull-down assays

Solid phase synthesis of the peptides was carried out on an Intavis Multipep Peptide Synthesizer (Intavis Bioanalytical Instruments AG) on a Rink amide LL resin, using N-Fmoc amino acids and HCTU as the coupling reagent. In the final steps of chain assembly biotin was incorporated at the N-terminal after an aminohexanoic acid spacer. Following this, the peptidylresin was added to 10 ml of 92.5% trifluoroacetic acid, 2.5% water, 2.5% ethanedithiol and 2.5% triisopropyl silane. After 2 h reaction, the resin was removed by filtration and the peptide was precipitated from the acid solution with diethyl ether on ice. The peptide was isolated by centrifugation, then dissolved in water and freeze dried overnight. After dissolving in water portions of the peptides were purified on a C8 reverse phase HPLC column (Agilent PrepHT Zorbax 300SB-C8, 21.2x250 mm, 7 m). Buffer A was 1% acetonitrile, 0.08% trifluoroacetic acid in water, buffer B was 90% acetonitrile, 0.08% trifluoroacetic acid in water. The elution gradient was from 10% to 50% B over 40 minutes at a flow rate of 8 ml/minute. The peak fraction was analysed by LC–MS on an Agilent 1100 LC-MSD. The calculated molecular weights of the peptide were in agreement with the mass found. For the complete list of peptides used in this study see Online Table IV.

Neutravidin^{TM} biotin binding beads (Perbio) were washed 3 times in 1 ml of cold Buffer Y [Tris-HCl pH 7.5 50 mM, NaCl 150 mM, EDTA 1 mM, NP-40 1 % v/v, DTT 1mM, EDTA-free Protease Inhibitor Cocktail (Sigma)] and incubated with the desired biotin-conjugated peptide (1.5 mg/ml in water) at 4°C for 1 h with rotation. Peptide-conjugated beads were washed 3 times in cold Buffer Y and incubated with 2 µg of purified protein in a volume of 500 µl of Buffer Y overnight at 4°C with rotation. Beads were then washed 3 times in Buffer Y and boiled in an appropriate volume of Laemmli buffer. Samples were analysed by standard Western blot analysis as described above.

TAT-peptide treatment

VEC-positive cells were plated at a concentration of 55.000 cells/cm^2 in complete culture medium with 30 µg/ml of TAT-conjugated peptide. Treatment was repeated the following day at the same concentration, followed by 2 other treatments at 10 µg/ml on day 3 and 5. On day 7, after cells had been at confluence for 48 h, cells were starved overnight in starving medium containing 10 µg/ml of TAT-conjugated peptide, then processed for co-immunoprecipitation, ChIP and qRT-PCR.

Chromatin immunoprecipitation (ChIP)

ChIP assays were carried out as previously described[20]. Briefly, cells were starved overnight and cross-linked with 1% PFA for 10 min at RT. PFA was then inactivated by the addition of 125 mM glycine. Cells were then washed and resuspended in lysis buffer. Samples were sonicated with a BIORUPTOR™ 200 using the following conditions: H power, 30 sec ON - 60 sec OFF for 20 min. Chromatin extracts containing from 200 μg to 1000 μg of DNA fragments with an average size of 500 bp were incubated overnight with using 5 μg of either immune antibody or matched non-immune antibodies and Dynabeads protein G (Invitrogen) or Ultralink resin protein A/G (Pierce ThermoScientific). On the following day, beads were recovered and washed twice with Mixed Micelle Wash Buffer (NaCl 150 mM, TrisHCl pH 8.1 20 mM, EDTA 5 mM, Sucrose 5.2 % w/v, NaN_3 0,02%, Triton X-100 1%, 0.2 % SDS), Buffer 500 (Deoxycholic acid 0,1% w/v, NaCl 500 mM, HEPES pH 7.5 25 mM, EDTA 1 mM, NaN_3 0.02%, Triton X-100 1%), LiCl Detergent wash (Deoxycholic acid 0,5% w/v, LiCl 250 mM, EDTA 1 mM, NP-40 0.5% v/v, NaN_3 0.02%, Tris HCl pH 8.0 10 mM). Proteins/DNA complexes were detached from beads by heating the samples at 65°C for 10 min. De-crosslinking was done at 65°C overnight. DNA was purified using phenol/chloroform and precipitated in 70% ethanol according to standard protocol. DNA was amplified by qRT-PCR techniques using oligonucleotides flanking the assayed promoter regions.

Primers used in this experiments were: 5'-GTCGGGTGAGCATTCAGTCT-3' and 5'-ATCAAGCCCACCCATCCTAC-3' (Claudin-5 TSS); 5'-TGCAGAAGGAGAAAACAATGC-3' and 5'-GCAGCAACGTGTGTCAGTGT-3' (VE-PTP Region 1); 5'-TGGATCCTGTAGCCATATTTGA-3' and 5'-CATCATATAACTGCAACAAAGCAC-3' (VE-PTP Region 2); 5'-GACATAAGTAGCCAAGAACAGGTTT-3' and 5'-TCAAATCACTAGGAGGAATAAGACA-3' (VE-PTP Region 3); 5'-GCTCAACAAGTGGTACCCAGA-3' and 5'-TGCACGACGCTCAGTGTTAT-3' (VE-PTP TSS); 5'-GTTTGTTTGAGCCAGGGTCT-3' and 5'-CAGGAGGTCGAAGCAAGATG-3' (vWf Region 1); 5'-GCAGGTCTTGGGTTCTATGC-3' and 5'-GGGGTGGAAATGATGGTTC-3' (vWf Region 2); 5'-TGGTGGCAACTTGGAGCTAT-3' and 5'-AGGGGCTTCAAAGTCCTCAG-3' (vWf Region 3/TSS).

For qRT-PCR analyses DNA was diluted in the presence of specific primers (0.4 μM each) to a final volume of 25 μl in SYBR Green Reaction Mix (Perkin Elmer).

RNA interference

Stealth RNAi Duplexes (Life Technologies) and the correspondent Low GC Stealth RNAi Control Duplexes (Life Technologies) were used to knockdown FoxO1. The sequences of the two siRNAs used were the following: 5'-CCAAGUGACUUGGAUGGCAUGUUUA-3' and 5'-CAGACACUUCAGGACAGCAAAUCAA-3'. Transfection was performed using LipofectAMINE 2000 (Invitrogen) according to the manufacturer's instructions.

***In vivo* pharmacological treatment**

All animal procedures were performed in accordance with the Institutional Animal Care and Use Committee (IACUC), in compliance with the guidelines established in the Principles of Laboratory Animal Care (directive 86/609/EEC) and approved by the Italian Ministry of Health.

Ezh2 and Ezh1 were inhibited in C57Bl/6J background pups by intraperitoneal injection with 50 mg/kg UNC1999[21] in 10% DMSO and 90% corn oil or vehicle only, at P3, P4 and P5. Total RNA from lungs of UNC1999-treated pups (P6) was isolated and processed as described in details previously[22].

Immunohistochemistry (IHC)

Paraffin-embedded human ovarian tissue samples were scored as pathologic or non-pathologic by a trained pathologist. Samples were de-paraffinized and hydrated following standard protocol and subjected to antigen unmasking in Sodium Citrate Buffer pH 6 for 45 min at 95°C. Samples were stained using MATCH 2 Double Stain 2 Kit (Biocare). Haematoxylin/eosin staining was performed according to standard protocol and samples were mounted in Eukitt (Bio-Optica).

For image quantification we used NIS-Elements AR software (version 4.51.01; Nikon). A hue saturation intensity (HSI) threshold was defined by selecting pixels of specific signal around the vessel perimeter. This threshold was applied to every image, followed by morphometrics steps of image smoothing, cleaning and filling. An area of interest was drawn on images to exclude regions of non-specific staining. Areas of detected specific signal were divided by the total measured surface, and values obtained for Claudin-5 and VE-cadherin were then normalized on the measurements obtained for PECAM1 in the corresponding regions.

Antibodies and chemicals

Antibodies used in this study were: anti-claudin-5 mouse monoclonal clone 4C3C2, anti-V5 tag mouse monoclonal R960-25 (Life Technologies); anti-claudin-5 rabbit polyclonal ab53765, anti-claudin-5 rabbit monoclonal ab131259, anti-CD31 rabbit polyclonal ab28364, anti-total histone H3 ab1791, anti-H3K4me3 ab8580, and anti-RNA pol II phospho Ser 5 (ABCAM); anti-β-catenin mouse monoclonal 610154, anti-Ezh2 mouse monoclonal 612666, anti-N-cadherin mouse monoclonal 610921, anti-Pecam1 rat monoclonal 550274 (BD Biosciences); anti-β-catenin rabbit polyclonal 06-734, anti-Ezh2 rabbit polyclonal 07-689, anti-H3K27me3 rabbit polyclonal 07-449, anti-TCF4 mouse monoclonal clone 6H5-3 05-511 (Millipore); anti-FKHR (H-130X) rabbit polyclonal sc-67140, anti-VE-cadherin goat polyclonal sc-6458, anti-vWf clone H-300 rabbit polyclonal sc-14014, anti-Suz12 goat polyclonal sc-46264 (Santa Cruz Biotechnology); anti-vWf rabbit polyclonal AB7356 (Chemicon); anti-FoxO1 rabbit monoclonal clone C29H4 2880, anti-phospho-FoxO1 (Ser 256) rabbit polyclonal 9461, anti-Myc-tag rabbit polyclonal 2272, anti-Suz12 rabbit monoclonal D39F6 3737, anti-H3K27me3 clone C36B11 rabbit monoclonal BK9733BFS, anti-β-catenin rabbit monoclonal 8480S (Cell Signalling), anti-Ezh2 mouse monoclonal NCL-L-EZH2 (Novocastra-Leica), peroxidase-conjugated streptavidin (Jackson ImmunoResearch Laboratories), anti-HA-tag mouse monoclonal clone 12CA5, anti-Bmi1 mouse monoclonal clone AF27 [from K. Helin, Biotech research and Innovation Centre (BRIC), University of Copenhagen], anti-Ezh2 clone AC22 and AE25-13, anti-α-tubulin mouse monoclonal, anti-vinculin mouse monoclonal (from internal service); anti-human VE-cadherin mouse monoclonal (BV9)[23], anti-VE-PTP Rabbit polyclonal (produced in our laboratory), anti-human VE-PTP Rabbit Polyclonal hPTP1-8 from D. Vestweber[24]; HRP-linked anti-GST RPN1236 (GE/Amersham); anti-VE-cadherin rabbit polyclonal 36-1900 (Thermofisher); anti-Ezh2 rabbit polyclonal 21800-1-AP (Proteintech).
The following reagents were used in this study: pan-caspase inhibitor Z-VAD-FMK (Promega); PI3K inhibitor LY294002 (Cell Signalling Technology). To inhibit PI3K activity, cells were starved for 24h in starving medium + 1% BSA and then treated overnight with LY294002 10 μM or Dimethyl sufoxide (DMSO) as a control. To avoid apoptotic cell death during FKHR-TM overexpression experiments, 24h after the beginning of the infection, cells were treated with Z-VAD-FMK 50 μM.

Statistical analysis

Student's two-tailed unpaired t-test was used to determine statistical significance. The significance level was set at $P < 0.05$.

ONLINE FIGURE LEGENDS

Online Figure I. VEC clustering induces *VE-PTP* and *vWf* expression through FoxO1/β-catenin inhibition.

(A) qRT-PCR and WB analysis of *VE-PTP* and *vWf* expression in sparse (sp) and confluent (con) VEC-null and VEC-positive ECs (upper panel). Immunofluorescence analysis of VEC (green) and *VE-PTP* or *vWf* expression (red) in confluent VEC-null and VEC-positive ECs. Scale bar: 10 μm (lower panel).
(B) qRT-PCR analysis of *VE-PTP* and *vWf* expression in VEC-null and VEC-positive ECs expressing FKHR-TM or GFP as a negative control. To limit the pro-apoptotic effect of FKHR-TM, after adenoviral infection the pan-caspase inhibitor Z-VAD-FMK (50 μM) was added to culture medium. qRT-PCR was performed 72 h after infection. The levels of mRNA are normalized to Glyceraldehyde-3-Phosphate Dehydrogenase (*GAPDH*), columns are means ± s.e.m. of triplicates from a typical experiment. nd, not detectable.
(C) qRT-PCR analysis of *VE-PTP* and *vWf* expression in confluent VEC-null and VEC-positive ECs treated with the PI3K inhibitor LY294002 (10 μM, overnight). Upper panel: WB analysis of p-FoxO1 Ser 256 (arrow) and total FoxO1 protein.
(D) qRT-PCR analysis of *VE-PTP* and *vWf* expression in confluent VEC-null and VEC-positive cells expressing ΔN-β-catenin or GFP as negative control.
(E) Schematic representation of the putative promoter regions of *VE-PTP* and *vWf*. FoxO1 (F) binding sites are depicted as white boxes and TCF/β catenin (T) binding sites as black boxes. Arrows identify primers used for qChIP shown in panels F and G.
(F) qRT-PCR for Region 1, 2 and 3 of *VE-PTP* and *vWf* promoter regions performed on endogenous FoxO1-bound chromatin immunoprecipitated from confluent VEC-null and VEC-positive ECs.

(G) qRT-PCR for Region 1, 2 and 3 of *VE-PTP* and *vWf* promoter regions performed on endogenous β-catenin-bound chromatin immunoprecipitated from confluent VEC-null and VEC-positive ECs.
In (A) and (C) vinculin was used as loading control.
In (A), (C) and (D) the levels of mRNA are normalized to 18S; columns are means ± s.e.m. of triplicates from a representative experiment.
In (B), (C) and (D) $^{**}P < 0.01$, *t*-test.
In (F) and (G) the levels of DNA are normalized to input, columns are means ± st.dev. of triplicates from a representative experiment. nd, not detectable. $^{*}P < 0.05$; $^{**}P < 0.01$, *t*-test VEC-null vs. VEC-positive.

Online Figure II. Validation of RNA-seq results.
(A) qRT-PCR analysis of *Tiam1*, *Lmo2*, *Stat6* and *Elk3* expression in confluent VEC-null and VEC-positive ECs.
(B) qRT-PCR analysis of *Hey1* and *Adm* expression in confluent VEC-null and VEC-positive ECs.
(C) qRT-PCR analysis of *Pecam1* and *Sox18* expression in confluent VEC-null and VEC-positive ECs.
(D) qRT-PCR analysis of *Claudin-5*, *VE-PTP* and *VWF* expression in sparse (sp) and confluent (con) HMEC1.
(E) qRT-PCR analysis of *TIAM1*, *LMO2*, *STAT6* and *ELK3* expression in sparse (sp) and confluent (con) HMEC1.
(F) qRT-PCR analysis of *HEY1* and *ADM* expression in sparse (sp) and confluent (con) HMEC1.
(G) qRT-PCR analysis of *PECAM1* and *SOX18* expression in sparse (sp) and confluent (con) HMEC1.
In the figure, the levels of mRNA are normalized to *GAPDH*; columns are means ± s.e.m. of triplicates from a representative experiment. $^{*}P < 0.05$; $^{**}P < 0.01$, *t*-test.

Online Figure III. *VE-PTP* and *vWf* genes are repressed in IL2-VEC ECs.
qRT-PCR analysis of *VE-PTP* and *vWf* expression in confluent VEC-null, VEC-positive and IL2-VEC ECs. The levels of mRNA are normalized to 18S; columns are means ± s.e.m. of triplicates from a representative experiment. $^{*}P < 0.01$, *t*-test.

Online Figure IV. Histone density on the TSS of *claudin-5, VE-PTP* and *vWf* is not affected by VEC expression.
qRT-PCR for the TSS of *claudin-5*, *VE-PTP* and *vWf* performed on endogenous histone H3-bound chromatin immunoprecipitated from confluent VEC-null and VEC-positive ECs. The levels of DNA are normalized to input, columns are means ± st.dev. of triplicates from a representative experiment.

Online Figure V. *Claudin-5* luciferase reporter assays.
(A) Schematic representation of the putative promoter region of *claudin-5* cloned upstream of firefly luciferase gene. FoxO1 (F) binding sites are depicted as white boxes and TCF/β catenin (T) binding sites as black boxes.
(B) *Claudin-5* luciferase reporter assay performed on confluent VEC-null and VEC-positive cells.
(C) *Claudin-5* luciferase reporter assay performed on confluent VEC-positive ECs upon Suz12 overexpression.
(D) *Claudin-5* luciferase reporter assay performed on confluent VEC-null ECs upon Suz12 knockdown (sh-Suz12).
In (B), (C) and (D) $^{*}P < 0.05$; $^{**}P < 0.01$, *t*-test.

Online Figure VI. Effect of Suz12 overexpression on endothelial genes.
(A) qRT-PCR analysis of *Lmo2* and *Stat6* expression in confluent VEC-positive ECs upon Suz12 overexpression.
(B) qRT-PCR analysis of *Hey1* expression in confluent VEC-positive ECs upon Suz12 overexpression.
(C) qRT-PCR analysis of *Pecam1* and *Sox18* expression in confluent VEC-positive ECs upon Suz12 overexpression.
In the figure, the levels of mRNA are normalized to *GAPDH*; columns are means ± s.e.m. of triplicates from a representative experiment. $^{*}P < 0.05$; $^{**}P < 0.01$, *t*-test.

Online Figure VII. FoxO1 mRNA levels are unaltered upon stable PcG protein knockdown.
qRT-PCR analysis of *FoxO1* expression in confluent VEC-null sh-Empty and VEC-null sh-Suz12 ECs. The levels of mRNA are normalized to 18S; columns are means ± s.e.m. of triplicates from a representative experiment.

Online Figure VIII. FoxO1 and Suz12 cooperate for repressing *claudin-5* expression.
(A) WB analysis of FKHR-TM, Suz12, H3K27me3 and claudin-5 in extracts of confluent VEC-null, VEC-positive-Empty and VEC-positive-Suz12 ECs expressing FKHR-TM or GFP (negative control). Tubulin is the loading control.
(B) qRT-PCR analysis of *claudin-5* expression in VEC-null, VEC-positive-Empty and VEC-positive-Suz12 ECs expressing FKHR-TM or GFP (negative control). Levels of mRNA are normalized to GAPDH. Columns are means ± s.e.m. of triplicates from a representative experiment. **P*<0.01, t-test.

Online Figure IX. Ezh2 peptide arrays.
Peptide arrays with overlapping 20-mers spanning the whole sequence of murine Ezh2 spotted on membrane, probed with indicated GST-tagged proteins in (B), (C) and (D) or free GST as control (A), and analysed by WB. Red dots identify peptides selected for streptavidin pull-down experiments shown in Figure 6. Green boxes define the areas shown in Figure 6B, 6F and 6L.

Online Figure X. Ezh2 interacts with p120-catenin.
Co-immunoprecipitation and WB of endogenous Ezh2 and p120-catenin from extracts of confluent VEC-null and VEC-positive ECs.

Online Figure XI. VE-PTP downregulation in vessels of serous surface papillary ovarian carcinoma.
IHC staining of VE-PTP expression in sections of human healthy ovary (left panel) or serous surface papillary ovarian carcinoma (right panel). Black arrowheads point to tumor vessel ECs expressing high levels of EZH2. Scale bar: 40 μm.

ONLINE TABLES

Online Table I. Significantly upregulated endothelial genes in VEC-positive vs VEC-null ECs. Complete list of endothelial genes significantly upregulated in VEC-positive cells according to the threshold |log2FC| ≥ 1 and adjusted p-value (padj) ≤ 0.05. List refers to Figure 1B.

Ensembl_gene_id	External_gene_name	log2FoldChange	padj
ENSMUSG00000021458	2010111I01Rik	1.030144555	0.000155219
ENSMUSG00000020681	Ace	1.080455751	2.39E-08
ENSMUSG00000054808	Actn4	1.571394864	5.72E-85
ENSMUSG00000000530	Acvrl1	3.011281082	3.07E-116
ENSMUSG00000022893	Adamts1	1.207992156	3.01E-34
ENSMUSG00000069833	Ahnak	1.002778723	4.12E-19
ENSMUSG00000019256	Ahr	2.306899238	8.88E-74
ENSMUSG00000066406	Akap13	1.147082083	3.31E-30
ENSMUSG00000021895	Arhgef3	1.557740572	2.19E-62
ENSMUSG00000055116	Arntl	1.63486021	1.38E-11
ENSMUSG00000051669	AU021092	2.631170051	4.87E-77
ENSMUSG00000037458	Azin1	1.694521168	9.20E-37
ENSMUSG00000049792	Bag5	1.037512828	5.20E-14
ENSMUSG00000059588	Calcrl	2.353845544	1.21E-91
ENSMUSG00000007655	Cav1	1.227512235	2.91E-32
ENSMUSG00000000058	Cav2	1.463840099	1.96E-19
ENSMUSG00000022661	Cd200	1.565319838	2.62E-12
ENSMUSG00000033502	Cdc14a	1.780455692	9.75E-33
ENSMUSG00000036533	Cdc42ep3	2.127347302	4.40E-35
ENSMUSG00000023067	Cdkn1a	1.582084046	2.75E-12
ENSMUSG00000071637	Cebpd	1.007602909	0.011257482
ENSMUSG00000041378	Cldn5	5.617650065	5.83E-76
ENSMUSG00000023959	Clic5	1.947174651	8.18E-105
ENSMUSG00000003617	Cp	3.425553469	3.73E-39
ENSMUSG00000006360	Crip1	4.100612414	8.93E-20
ENSMUSG00000061353	Cxcl12	1.61906941	4.58E-11
ENSMUSG00000028195	Cyr61	1.614984821	1.58E-10
ENSMUSG00000022150	Dab2	1.108024746	5.42E-21
ENSMUSG00000002257	Def6	2.77107749	1.67E-11
ENSMUSG00000040631	Dok4	1.004119184	1.66E-14
ENSMUSG00000003518	Dusp3	1.335031683	3.23E-57
ENSMUSG00000028108	Ecm1	1.087583892	2.06E-12
ENSMUSG00000008398	Elk3	1.080804982	2.57E-37
ENSMUSG00000022505	Emp2	1.577772246	8.46E-12
ENSMUSG00000024140	Epas1	1.005688736	1.04E-33
ENSMUSG00000038776	Ephx1	1.351199263	1.93E-14
ENSMUSG00000024778	Fas	4.169362351	4.39E-36
ENSMUSG00000003420	Fcgrt	1.978980212	6.33E-51
ENSMUSG00000040170	Fmo2	2.04526583	0.000500035

ENSMUSG00000009687	Fxyd5	1.339637934	8.36E-24
ENSMUSG00000015312	Gadd45b	1.483447133	0.000612635
ENSMUSG00000034201	Gas2l1	1.006587513	1.30E-09
ENSMUSG00000021360	Gcnt2	1.904024924	2.17E-15
ENSMUSG00000055737	Ghr	1.411347191	3.38E-16
ENSMUSG00000054435	Gimap4	2.783636744	4.28E-105
ENSMUSG00000050105	Grrp1	1.129108898	0.025674785
ENSMUSG00000040562	Gstm2	2.083053527	1.45E-12
ENSMUSG00000067212	H2-T23	1.48958506	3.67E-20
ENSMUSG00000071379	Hpcal1	1.160288402	4.98E-09
ENSMUSG00000003541	Ier3	2.349737755	2.52E-10
ENSMUSG00000074896	Ifit3	1.814510316	3.07E-15
ENSMUSG00000005533	Igf1r	1.840922626	0.000836188
ENSMUSG00000022969	Il10rb	1.029032008	4.36E-10
ENSMUSG00000017057	Il13ra1	1.234914814	0.036664413
ENSMUSG00000018899	Irf1	1.203207384	6.74E-14
ENSMUSG00000027276	Jag1	1.101653799	6.97E-17
ENSMUSG00000021294	Kif26a	1.432481827	1.15E-15
ENSMUSG00000031788	Kifc3	1.976925202	9.04E-28
ENSMUSG00000055148	Klf2	1.488630726	1.41E-18
ENSMUSG00000003032	Klf4	2.801834854	6.83E-94
ENSMUSG00000033863	Klf9	2.562506604	2.76E-36
ENSMUSG00000021959	Lats2	1.193141326	5.64E-25
ENSMUSG00000054263	Lifr	1.084566843	2.40E-06
ENSMUSG00000032698	Lmo2	1.511260593	5.22E-37
ENSMUSG00000016520	Lnx2	1.290227959	1.83E-20
ENSMUSG00000068015	Lrch1	1.419034427	7.97E-37
ENSMUSG00000040488	Ltbp4	1.12334367	8.90E-07
ENSMUSG00000001089	Luzp1	1.028630163	5.55E-19
ENSMUSG00000075602	Ly6a	1.812932107	1.17E-16
ENSMUSG00000022587	Ly6e	1.028516047	2.27E-11
ENSMUSG00000042622	Maff	1.194577554	0.017765841
ENSMUSG00000028862	Map3k6	1.348510986	0.038153326
ENSMUSG00000001493	Meox1	4.746823504	1.38E-22
ENSMUSG00000022353	Mtss1	1.425709869	1.67E-56
ENSMUSG00000037235	Mxd4	1.105213479	3.24E-35
ENSMUSG00000021365	Nedd9	1.012790987	7.52E-23
ENSMUSG00000003847	Nfat5	1.085110516	2.67E-30
ENSMUSG00000021025	Nfkbia	1.26736757	5.21E-09
ENSMUSG00000021806	Nid2	2.24487386	1.32E-126
ENSMUSG00000020889	Nr1d1	2.192153131	2.56E-11
ENSMUSG00000020019	Ntn4	1.060251028	2.58E-13
ENSMUSG00000022146	Osmr	2.214586778	5.94E-05
ENSMUSG00000024725	Ostf1	1.483312676	2.43E-32
ENSMUSG00000024805	Pcgf5	1.427740658	0.000138545
ENSMUSG00000051177	Plcb1	2.625979803	3.78E-89

ENSMUSG00000032377	Plscr4	2.938641363	1.61E-09
ENSMUSG00000017754	Pltp	3.603782243	4.70E-44
ENSMUSG00000047714	Ppp1r2	1.133018396	2.00E-41
ENSMUSG00000053198	Prx	1.312060881	4.93E-07
ENSMUSG00000027864	Ptgfrn	1.760648665	0.001114651
ENSMUSG00000059895	Ptp4a3	1.076091502	4.20E-14
ENSMUSG00000020154	Ptprb	1.870491603	2.59E-102
ENSMUSG00000020151	Ptprr	3.906271548	1.03E-14
ENSMUSG00000009291	Pttg1ip	1.494446887	6.28E-54
ENSMUSG00000062232	Rapgef2	1.384227356	1.15E-36
ENSMUSG00000054364	Rhob	1.181079153	3.54E-19
ENSMUSG00000033107	Rnf125	1.575761287	1.32E-12
ENSMUSG00000021067	Sav1	1.563868928	3.56E-41
ENSMUSG00000028780	Sema3c	1.166899613	0.006795405
ENSMUSG00000071178	Serpina1b	4.035515589	4.23E-19
ENSMUSG00000071177	Serpina1d	3.738797408	3.27E-13
ENSMUSG00000072849	Serpina1e	3.335872183	3.95E-10
ENSMUSG00000017756	Slc12a7	2.252879677	5.02E-69
ENSMUSG00000037434	Slc30a1	1.178611424	2.16E-21
ENSMUSG00000025993	Slc40a1	1.018321315	5.11E-10
ENSMUSG00000030096	Slc6a6	1.770344183	2.31E-17
ENSMUSG00000002504	Slc9a3r2	1.286876353	7.09E-34
ENSMUSG00000032548	Slco2a1	2.471737479	6.58E-10
ENSMUSG00000025006	Sorbs1	1.153251032	5.20E-14
ENSMUSG00000031626	Sorbs2	1.075504026	3.75E-14
ENSMUSG00000002147	Stat6	1.511251856	4.96E-28
ENSMUSG00000014813	Stc1	1.078916739	8.98E-40
ENSMUSG00000039156	Stim2	1.240683389	1.07E-45
ENSMUSG00000030711	Sult1a1	1.055824496	0.014286389
ENSMUSG00000096054	Syne1	1.043358879	1.65E-14
ENSMUSG00000038213	Tapbpl	1.210269439	3.14E-06
ENSMUSG00000006642	Tcf23	2.402352937	1.27E-08
ENSMUSG00000020034	Tcp11l2	1.137117364	1.82E-08
ENSMUSG00000002489	Tiam1	2.185948789	4.27E-102
ENSMUSG00000020044	Timp3	1.710305929	9.82E-89
ENSMUSG00000034640	Tiparp	3.307295131	3.95E-127
ENSMUSG00000020023	Tmcc3	2.227468759	9.42E-09
ENSMUSG00000037573	Tob1	1.334672963	6.87E-13
ENSMUSG00000020601	Trib2	1.368183264	4.98E-15
ENSMUSG00000047821	Trim16	1.05040397	5.32E-08
ENSMUSG00000020773	Trim47	1.808934868	3.01E-18
ENSMUSG00000042116	Vwa1	4.388395603	7.03E-88
ENSMUSG00000001930	Vwf	2.10472827	3.69E-88
ENSMUSG00000044786	Zfp36	1.277531768	0.007208359
ENSMUSG00000041703	Zic5	1.557135924	0.011888894

Online Table II. Significantly downregulated endothelial genes in VEC-positive vs VEC-null ECs. Complete list of endothelial genes significantly downregulated in VEC-positive cells according to the threshold |log2FC| ≥ 1 and adjusted p-value (padj) ≤ 0.05. List refers to Figure 1B.

Ensembl_gene_id	External_gene_name	log2FoldChange	padj
ENSMUSG00000025085	Ablim1	-1.945664238	5.37E-24
ENSMUSG00000026003	Acadl	-1.086609504	2.58E-10
ENSMUSG00000030790	Adm	-1.608017282	0.010413999
ENSMUSG00000038587	Akap12	-1.726967454	2.61E-34
ENSMUSG00000041688	Amot	-1.758695706	2.43E-32
ENSMUSG00000071847	Apcdd1	-1.149995659	0.001802338
ENSMUSG00000027792	Bche	-1.514352417	0.013541964
ENSMUSG00000026278	Bok	-1.289180293	0.000255985
ENSMUSG00000026029	Casp8	-1.067999624	2.80E-19
ENSMUSG00000041598	Cdc42ep4	-1.048941056	0.008913035
ENSMUSG00000037664	Cdkn1c	-1.557952614	4.96E-06
ENSMUSG00000052560	Cpne8	-2.080693021	6.82E-28
ENSMUSG00000044258	Ctla2a	-1.245822534	7.41E-19
ENSMUSG00000074874	Ctla2b	-1.670778565	6.31E-25
ENSMUSG00000019891	Dcbld1	-1.138077823	0.004201829
ENSMUSG00000057098	Ebf1	-2.111413582	9.99E-12
ENSMUSG00000027954	Efna1	-1.200342638	1.03E-18
ENSMUSG00000041773	Enc1	-1.79080039	1.66E-23
ENSMUSG00000043556	Fbxl7	-3.775920835	5.86E-14
ENSMUSG00000038372	Gmds	-1.004691345	0.004715295
ENSMUSG00000056870	Gulp1	-1.419817989	0.020827657
ENSMUSG00000040289	Hey1	-2.020566228	6.67E-08
ENSMUSG00000007872	Id3	-1.394713645	2.94E-07
ENSMUSG00000026896	Ifih1	-1.325927571	0.010695402
ENSMUSG00000078853	Igtp	-1.198266255	0.020467277
ENSMUSG00000031304	Il2rg	-3.162676661	2.01E-14
ENSMUSG00000031734	Irx3	-2.38750018	6.26E-20
ENSMUSG00000031239	Itm2a	-1.759963614	4.14E-06
ENSMUSG00000098557	Kctd12	-1.851946008	1.84E-06
ENSMUSG00000057722	Lepr	-1.490989244	0.005647257
ENSMUSG00000018169	Mfng	-2.644030792	9.44E-15
ENSMUSG00000023094	Msrb2	-1.333390874	0.000634309
ENSMUSG00000020900	Myh10	-1.492692468	1.59E-35
ENSMUSG00000018417	Myo1b	-3.305487303	1.90E-133
ENSMUSG00000046949	Nqo2	-1.085224089	1.64E-09
ENSMUSG00000033377	Palmd	-1.624208436	0.004043486
ENSMUSG00000031379	Pir	-4.724267197	1.95E-22
ENSMUSG00000042842	Serpinb6b	-1.942757029	1.63E-06
ENSMUSG00000045629	Sh3tc2	-6.052872043	1.87E-43
ENSMUSG00000040710	St8sia4	-2.233886401	7.77E-46
ENSMUSG00000037820	Tgm2	-5.967240498	4.34E-191

ENSMUSG00000060548	Tnfrsf19	-1.869105773	2.51E-11
ENSMUSG00000020577	Tspan13	-5.490186323	1.60E-39
ENSMUSG00000001473	Tubb6	-1.290729983	4.37E-30

Online Table III. Grouping of functional annotation categories.
Explanation of how different annotation terms with overlapping gene sets were grouped in Figure 1C graphical representation.

Direction	Group	Group in Figure 1C
down	UP_KEYWORDS: Cell cycle	cell cycle
down	GOTERM_BP_DIRECT: GO:0007049~cell cycle	cell cycle
down	UP_KEYWORDS: Cytoskeleton	cytoskeleton
down	UP_KEYWORDS: Phosphoprotein	phosphoprotein / signal
down	GOTERM_BP_DIRECT: GO:0007052~mitotic spindle organization	mitotic spindle organization
down	UP_KEYWORDS: Cell projection	cell projection
down	UP_KEYWORDS: Cyclin	cell proliferation / cyclin
down	GOTERM_BP_DIRECT: GO:0008283~cell proliferation	cell proliferation / cyclin
down	GOTERM_BP_DIRECT: GO:0002040~sprouting angiogenesis	sprouting angiogenesis
up	UP_KEYWORDS: Glycoprotein	glycoprotein
up	UP_KEYWORDS: Disulfide bond	disulfide bond
up	UP_KEYWORDS: Signal	phosphoprotein / signal
up	KEGG_PATHWAY: mmu04510:Focal adhesion	ECM-receptor interaction / focal adhesion
up	KEGG_PATHWAY: mmu04151:PI3K-Akt signaling pathway	PI3K-AKT signaling pathway
up	KEGG_PATHWAY: mmu04512:ECM-receptor interaction	ECM-receptor interaction / focal adhesion
up	UP_KEYWORDS: Developmental protein	developmental protein / differentiation
up	UP_KEYWORDS: Differentiation	developmental protein / differentiation
up	UP_KEYWORDS: Membrane	membrane / transmembrane
up	UP_KEYWORDS: Transmembrane	membrane / transmembrane
up	UP_KEYWORDS: Protein phosphatase	protein phosphatase / transferase
up	UP_KEYWORDS: Kinase	kinase
up	UP_KEYWORDS: Transferase	protein phosphatase / transferase
up	UP_KEYWORDS: Serine/threonine-protein kinase	kinase
up	BIOCARTA: m_raccycdPathway:Influence of Ras and Rho proteins on G1 to S Transition	influence of Ras and Rho proteins on G1 to S transition
up	UP_KEYWORDS: Cell cycle	cell cycle

Online Table IV. Complete list of peptides used in this study.

Biotinylated peptides used in pull-down assays	
A2	Biotin-X-G-Q-T-G-K-K-S-E-K-G-P-V-C-W-R-K-R-V-K-S-CONH2
A18	Biotin-X-R-V-K-S-E-Y-M-R-L-R-Q-L-K-R-F-R-R-A-D-E-CONH2
B33	Biotin-X-I-M-T-S-V-S-S-L-R-G-T-R-E-C-S-V-T-S-D-L-CONH2
C2	Biotin-X-S-L-R-G-T-R-E-C-S-V-T-S-D-L-D-F-P-A-Q-V-CONH2
E12	Biotin-X-D-R-E-C-G-F-I-N-D-E-I-F-V-E-L-V-N-A-L-G-CONH2
F10	Biotin-X-E-R-E-E-K-Q-K-D-L-E-D-N-R-D-D-K-E-T-C-P-CONH2
H34	Biotin-X-D-C-F-L-H-P-F-H-A-T-P-N-T-Y-K-R-K-N-T-E-CONH2
I9	Biotin-X-T-Y-K-R-K-N-T-E-T-A-L-D-N-K-P-C-G-P-Q-C-CONH2
I27	Biotin-X-Q-C-Y-Q-H-L-E-G-A-K-E-F-A-A-A-L-T-A-E-R-CONH2
K31	Biotin-X-K-D-E-T-S-S-S-S-E-A-N-S-R-C-Q-T-P-I-K-M-CONH2
K35	Biotin-X-S-S-S-S-E-A-N-S-R-C-Q-T-P-I-K-M-K-P-N-I-CONH2
L4	Biotin-X-N-S-R-C-Q-T-P-I-K-M-K-P-N-I-E-P-P-E-N-V-CONH2
L5	Biotin-X-S-R-C-Q-T-P-I-K-M-K-P-N-I-E-P-P-E-N-V-E-CONH2
L7	Biotin-X-C-Q-T-P-I-K-M-K-P-N-I-E-P-P-E-N-V-E-W-S-CONH2
M6	Biotin-X-N-F-C-A-I-A-R-L-I-G-T-K-T-C-R-Q-V-Y-E-F-CONH2
M10	Biotin-X-I-A-R-L-I-G-T-K-T-C-R-Q-V-Y-E-F-R-V-K-E-CONH2
M17	Biotin-X-K-T-C-R-Q-V-Y-E-F-R-V-K-E-S-S-I-I-A-P-V-CONH2
M26	Biotin-X-R-V-K-E-S-S-I-I-A-P-V-P-T-E-D-V-D-T-P-P-CONH2
N3	Biotin-X-D-V-D-T-P-P-R-K-K-K-R-K-H-R-L-W-A-A-H-C-CONH2
N22	Biotin-X-C-R-K-I-Q-L-K-K-D-G-S-S-N-H-V-Y-N-Y-Q-P-CONH2
N35	Biotin-X-H-V-Y-N-Y-Q-P-C-D-H-P-R-Q-P-C-D-S-S-C-P-CONH2
O4	Biotin-X-P-C-D-H-P-R-Q-P-C-D-S-S-C-P-C-V-I-A-Q-N-CONH2
O25	Biotin-X-C-E-K-F-C-Q-C-S-S-E-C-Q-N-R-F-P-G-C-R-C-CONH2
O28	Biotin-X-F-C-Q-C-S-S-E-C-Q-N-R-F-P-G-C-R-C-K-A-Q-CONH2
O35	Biotin-X-C-Q-N-R-F-P-G-C-R-C-K-A-Q-C-N-T-K-Q-C-P-CONH2
P2	Biotin-X-F-P-G-C-R-C-K-A-Q-C-N-T-K-Q-C-P-C-Y-L-A-CONH2
P30	Biotin-X-C-L-T-C-G-A-A-D-H-W-D-S-K-N-V-S-C-K-N-C-CONH2
R8	Biotin-X-F-I-S-E-Y-C-G-E-I-I-S-Q-D-E-A-D-R-R-G-K-CONH2
R23	Biotin-X-D-R-R-G-K-V-Y-D-K-Y-M-C-S-F-L-F-N-L-N-N-CONH2
S4	Biotin-X-N-N-D-F-V-V-D-A-T-R-K-G-N-K-I-R-F-A-N-H-CONH2
S10	Biotin-X-D-A-T-R-K-G-N-K-I-R-F-A-N-H-S-V-N-P-N-C-CONH2
S22	Biotin-X-N-H-S-V-N-P-N-C-Y-A-K-V-M-M-V-N-G-D-H-R-CONH2
T11	Biotin-X-R-A-I-Q-T-G-E-E-L-F-F-D-Y-R-Y-S-Q-A-D-A-CONH2
ctr-D28	Biotin-X-L-D-Q-D-G-T-F-I-E-E-L-I-K-N-Y-D-G-K-V-H-CONH2
ctr-G5	Biotin-X-I-S-S-M-F-P-D-K-G-T-A-E-E-L-K-E-K-Y-K-E-CONH2
ctr-K11	Biotin-X-D-R-E-A-G-T-E-T-G-G-E-N-N-D-K-E-E-E-E-K-CONH2
TAT-peptides used in VEC-positive EC treatment	
TAT-M6	Biotin-X-N-F-C-A-I-A-R-L-I-G-T-K-T-C-R-Q-V-Y-E-F -YGRKKRRQRRR-CONH2
TAT-M10	Biotin-X-I-A-R-L-I-G-T-K-T-C-R-Q-V-Y-E-F-R-V-K-E-YGRKKRRQRRR-CONH2
TAT-O4	Biotin-X-P-C-D-H-P-R-Q-P-C-D-S-S-C-P-C-V-I-A-Q-N-YGRKKRRQRRR-CONH2
TAT-P30	Biotin-X-C-L-T-C-G-A-A-D-H-W-D-S-K-N-V-S-C-K-N-C-YGRKKRRQRRR-CONH2
TAT-ctr-K11	Biotin-X-D-R-E-A-G-T-E-T-G-G-E-N-N-D-K-E-E-E-E-K-YGRKKRRQRRR-CONH2

SUPPLEMENTAL REFERENCES

1. Balconi G, Spagnuolo R, Dejana E. Development of endothelial cell lines from embryonic stem cells: A tool for studying genetically manipulated endothelial cells in vitro. *Arteriosclerosis, thrombosis, and vascular biology*. 2000;20:1443-1451
2. Grazia Lampugnani M, Zanetti A, Corada M, Takahashi T, Balconi G, Breviario F, Orsenigo F, Cattelino A, Kemler R, Daniel TO, Dejana E. Contact inhibition of vegf-induced proliferation requires vascular endothelial cadherin, beta-catenin, and the phosphatase dep-1/cd148. *The Journal of cell biology*. 2003;161:793-804
3. Navarro P, Caveda L, Breviario F, Mandoteanu I, Lampugnani MG, Dejana E. Catenin-dependent and -independent functions of vascular endothelial cadherin. *The Journal of biological chemistry*. 1995;270:30965-30972
4. Lampugnani MG, Zanetti A, Breviario F, Balconi G, Orsenigo F, Corada M, Spagnuolo R, Betson M, Braga V, Dejana E. Ve-cadherin regulates endothelial actin activating rac and increasing membrane association of tiam. *Molecular biology of the cell*. 2002;13:1175-1189
5. Xiao K, Allison DF, Kottke MD, Summers S, Sorescu GP, Faundez V, Kowalczyk AP. Mechanisms of ve-cadherin processing and degradation in microvascular endothelial cells. *The Journal of biological chemistry*. 2003;278:19199-19208
6. Cattelino A, Liebner S, Gallini R, Zanetti A, Balconi G, Corsi A, Bianco P, Wolburg H, Moore R, Oreda B, Kemler R, Dejana E. The conditional inactivation of the beta-catenin gene in endothelial cells causes a defective vascular pattern and increased vascular fragility. *The Journal of cell biology*. 2003;162:1111-1122
7. Bussolino F, De Rossi M, Sica A, Colotta F, Wang JM, Bocchietto E, Padura IM, Bosia A, DeJana E, Mantovani A. Murine endothelioma cell lines transformed by polyoma middle t oncogene as target for and producers of cytokines. *Journal of immunology*. 1991;147:2122-2129
8. Dull T, Zufferey R, Kelly M, Mandel RJ, Nguyen M, Trono D, Naldini L. A third-generation lentivirus vector with a conditional packaging system. *Journal of virology*. 1998;72:8463-8471
9. Daly C, Wong V, Burova E, Wei Y, Zabski S, Griffiths J, Lai KM, Lin HC, Ioffe E, Yancopoulos GD, Rudge JS. Angiopoietin-1 modulates endothelial cell function and gene expression via the transcription factor fkhr (foxo1). *Genes & development*. 2004;18:1060-1071
10. Quasnichka H, Slater SC, Beeching CA, Boehm M, Sala-Newby GB, George SJ. Regulation of smooth muscle cell proliferation by beta-catenin/t-cell factor signaling involves modulation of cyclin d1 and p21 expression. *Circulation research*. 2006;99:1329-1337
11. Lampugnani MG, Corada M, Andriopoulou P, Esser S, Risau W, Dejana E. Cell confluence regulates tyrosine phosphorylation of adherens junction components in endothelial cells. *Journal of cell science*. 1997;110 (Pt 17):2065-2077
12. Kim D, Pertea G, Trapnell C, Pimentel H, Kelley R, Salzberg SL. Tophat2: Accurate alignment of transcriptomes in the presence of insertions, deletions and gene fusions. *Genome Biol*. 2013;14:R36
13. Durinck S, Moreau Y, Kasprzyk A, Davis S, De Moor B, Brazma A, Huber W. Biomart and bioconductor: A powerful link between biological databases and microarray data analysis. *Bioinformatics*. 2005;21:3439-3440
14. Love MI, Huber W, Anders S. Moderated estimation of fold change and dispersion for rna-seq data with deseq2. *Genome Biol*. 2014;15:550
15. Huang da W, Sherman BT, Lempicki RA. Systematic and integrative analysis of large gene lists using david bioinformatics resources. *Nat Protoc*. 2009;4:44-57
16. Spagnuolo R, Corada M, Orsenigo F, Zanetta L, Deuschle U, Sandy P, Schneider C, Drake CJ, Breviario F, Dejana E. Gas1 is induced by ve-cadherin and vascular endothelial growth factor and inhibits endothelial cell apoptosis. *Blood*. 2004;103:3005-3012
17. Cartharius K, Frech K, Grote K, Klocke B, Haltmeier M, Klingenhoff A, Frisch M, Bayerlein M, Werner T. Matinspector and beyond: Promoter analysis based on transcription factor binding sites. *Bioinformatics*. 2005;21:2933-2942
18. Taddei A, Giampietro C, Conti A, Orsenigo F, Breviario F, Pirazzoli V, Potente M, Daly C, Dimmeler S, Dejana E. Endothelial adherens junctions control tight junctions by ve-cadherin-mediated upregulation of claudin-5. *Nature cell biology*. 2008;10:923-934

19. Giampietro C, Disanza A, Bravi L, Barrios-Rodiles M, Corada M, Frittoli E, Savorani C, Lampugnani MG, Boggetti B, Niessen C, Wrana JL, Scita G, Dejana E. The actin-binding protein eps8 binds ve-cadherin and modulates yap localization and signaling. *J Cell Biol*. 2015;211:1177-1192
20. Nakae J, Kitamura T, Kitamura Y, Biggs WH, 3rd, Arden KC, Accili D. The forkhead transcription factor foxo1 regulates adipocyte differentiation. *Developmental cell*. 2003;4:119-129
21. Konze KD, Ma A, Li F, Barsyte-Lovejoy D, Parton T, Macnevin CJ, Liu F, Gao C, Huang XP, Kuznetsova E, Rougie M, Jiang A, Pattenden SG, Norris JL, James LI, Roth BL, Brown PJ, Frye SV, Arrowsmith CH, Hahn KM, Wang GG, Vedadi M, Jin J. An orally bioavailable chemical probe of the lysine methyltransferases ezh2 and ezh1. *ACS chemical biology*. 2013;8:1324-1334
22. Corada M, Orsenigo F, Morini MF, Pitulescu ME, Bhat G, Nyqvist D, Breviario F, Conti V, Briot A, Iruela-Arispe ML, Adams RH, Dejana E. Sox17 is indispensable for acquisition and maintenance of arterial identity. *Nature communications*. 2013;4:2609
23. Corada M, Liao F, Lindgren M, Lampugnani MG, Breviario F, Frank R, Muller WA, Hicklin DJ, Bohlen P, Dejana E. Monoclonal antibodies directed to different regions of vascular endothelial cadherin extracellular domain affect adhesion and clustering of the protein and modulate endothelial permeability. *Blood*. 2001;97:1679-1684
24. Hayashi M, Majumdar A, Li X, Adler J, Sun Z, Vertuani S, Hellberg C, Mellberg S, Koch S, Dimberg A, Koh GY, Dejana E, Belting HG, Affolter M, Thurston G, Holmgren L, Vestweber D, Claesson-Welsh L. Ve-ptp regulates vegfr2 activity in stalk cells to establish endothelial cell polarity and lumen formation. *Nature communications*. 2013;4:1672

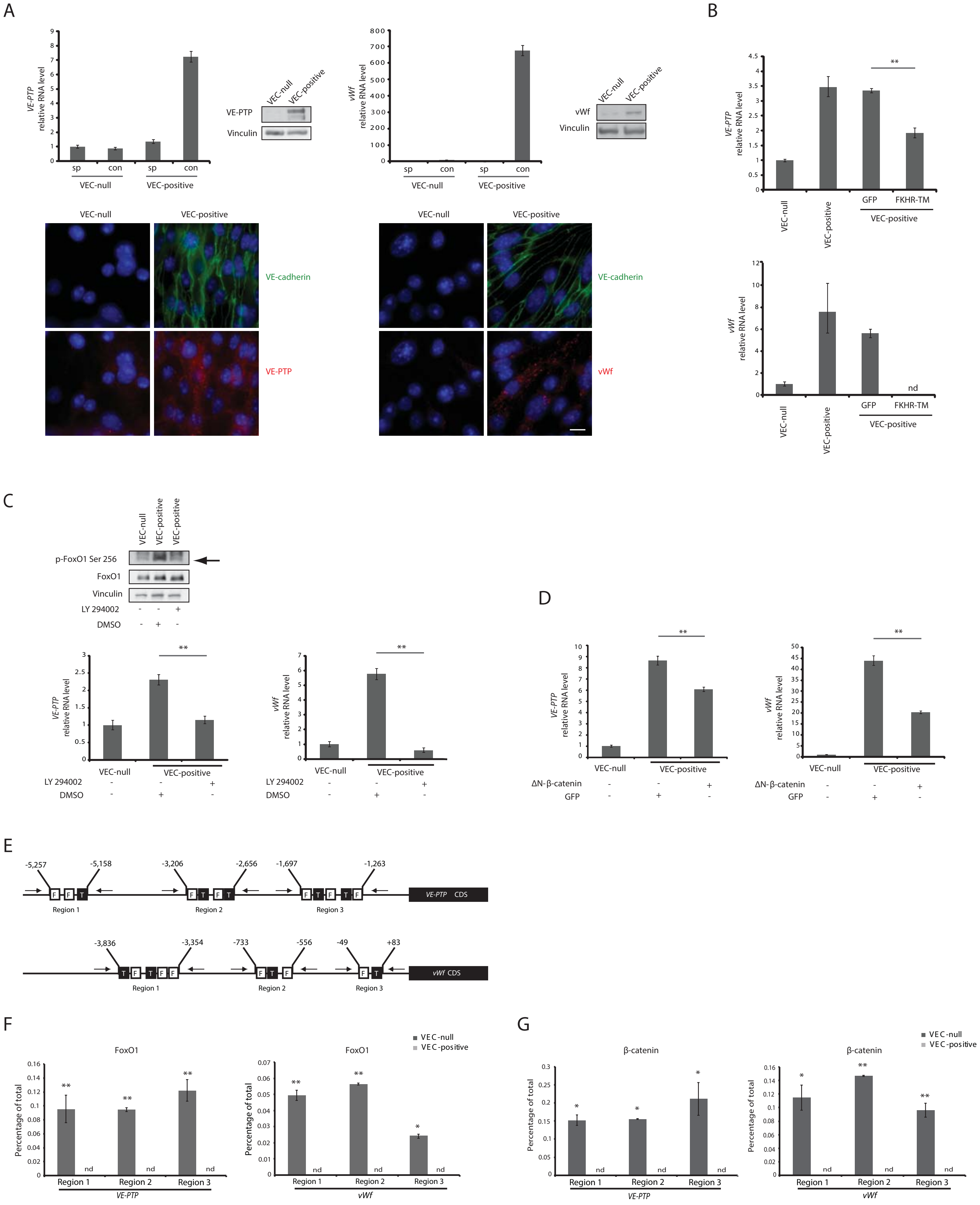

Online Figure I

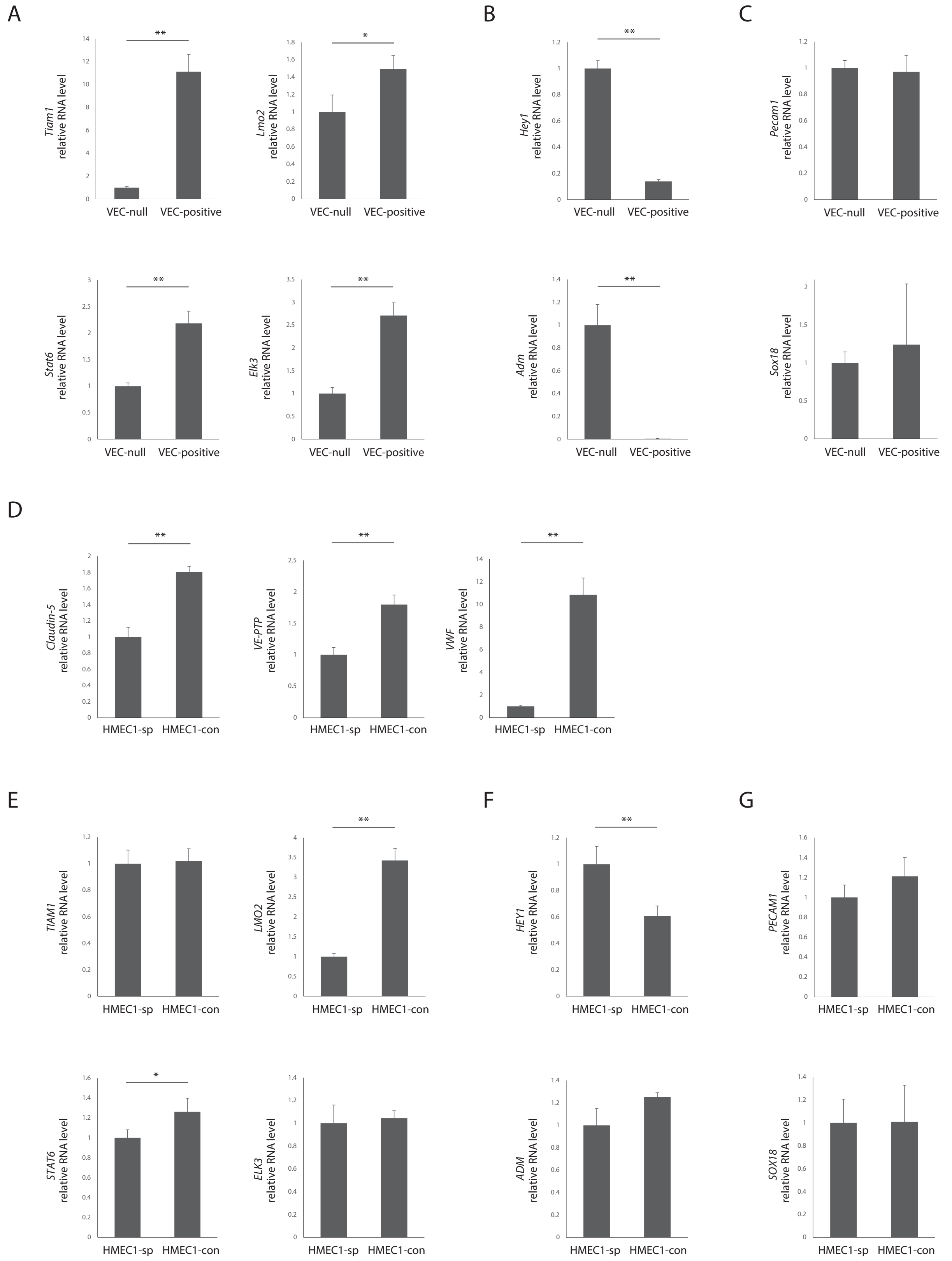

Online Figure II

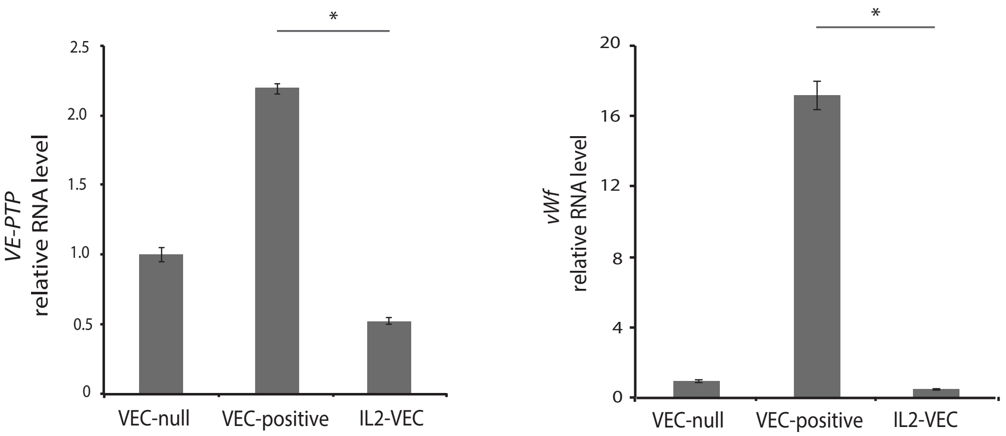

Online Figure III

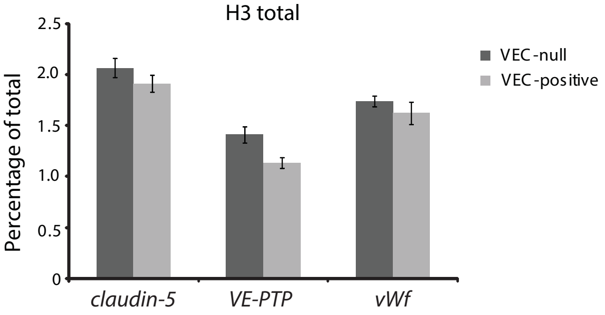

Online Figure IV

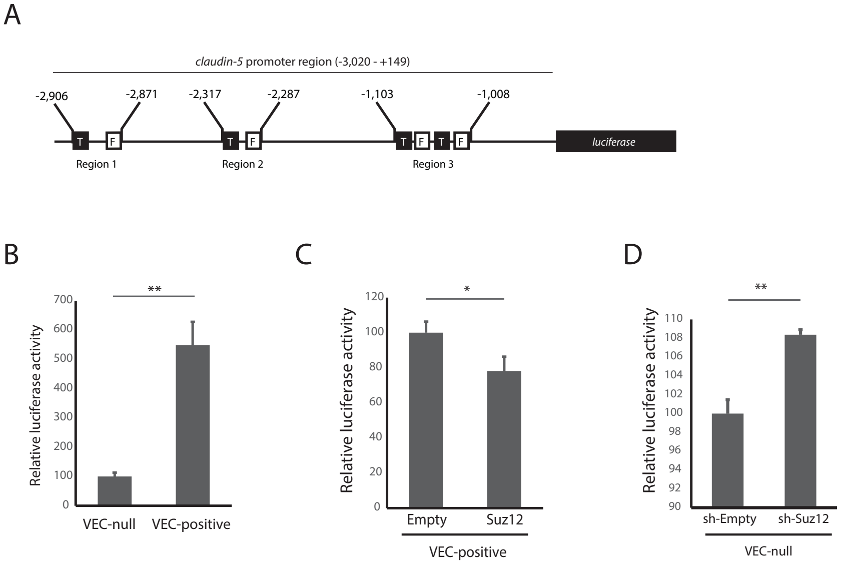

Online Figure V

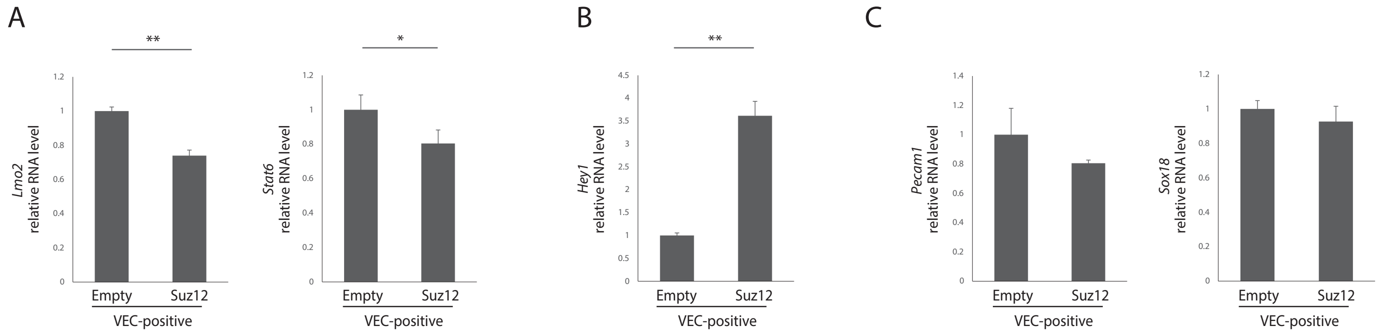

Online Figure VI

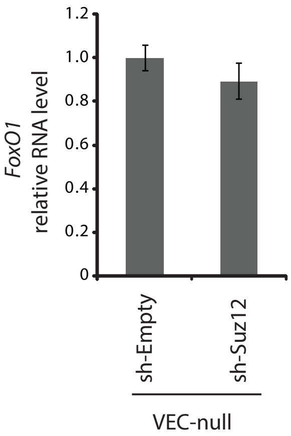

Online Figure VII

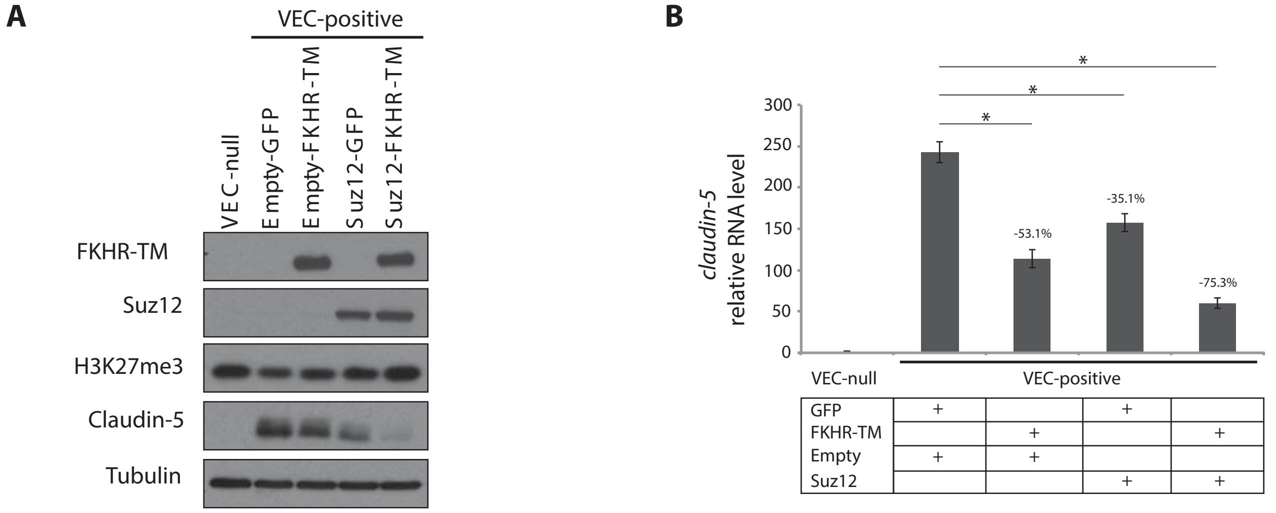

Online Figure VIII

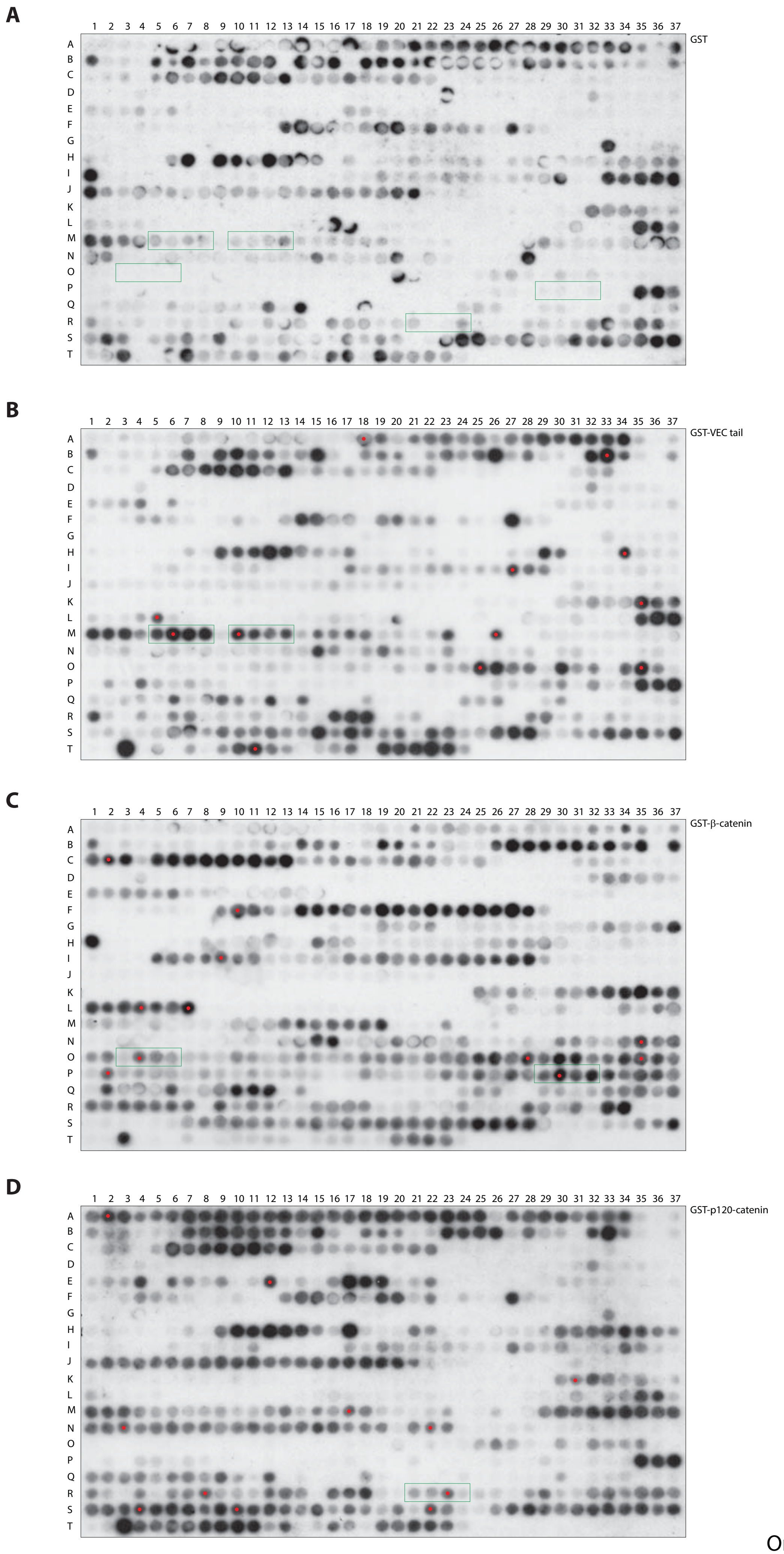

Online Figure IX

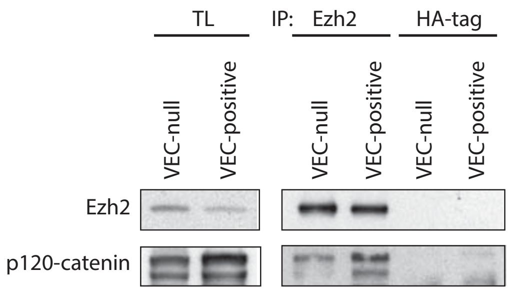

Online Figure X

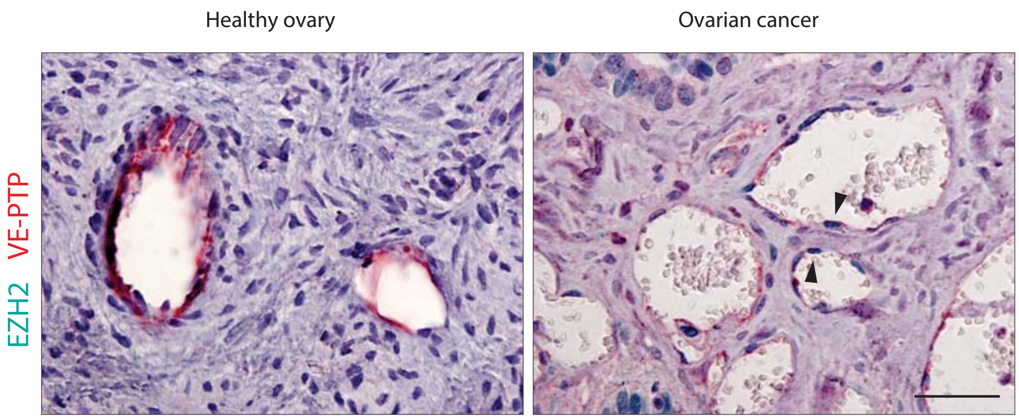

Online Figure XI
